# Mylotarg has potent anti-leukaemic effect: a systematic review and meta-analysis of anti-CD33 antibody treatment in acute myeloid leukaemia

**DOI:** 10.1007/s00277-014-2218-6

**Published:** 2014-10-05

**Authors:** J. Loke, J. N. Khan, J. S. Wilson, C. Craddock, K. Wheatley

**Affiliations:** 1 Centre for Clinical Haematology, Queen Elizabeth Hospital, Birmingham, UK; 2 Warwick Medical School, University of Warwick, Coventry, CV4 7AL UK; 3 Cancer Research UK Clinical Trials Unit (CRCTU), University of Birmingham, Edgbaston, Birmingham, UK

**Keywords:** Acute myeloid leukaemia, CD33 antigen, Systematic review, Meta-analysis, Randomised clinical trials, Gemtuzumab, Mylotarg, Humanised monoclonal antibodies

## Abstract

Conventional chemotherapy is ineffective in the majority of patients with acute myeloid leukaemia (AML), and monoclonal antibodies recognising CD33 expressed on myeloid progenitors (e.g. gemtuzumab ozogamicin (GO)) have been reported to improve outcome in patients with AML. Reports of excess toxicity have resulted in GO’s licence being withdrawn. As a result, the role of these agents remains unclear. A systematic review and meta-analysis included studies of patients with AML who had entered a randomised control trial (RCT), where one arm included anti-CD33 antibody therapy. Fixed effect meta-analysis was used, involving calculation of observed minus expected number of events, and variance for each endpoint in each trial, with the overall treatment effect expressed as Peto’s odds ratio with 95 % confidence interval. Meta-analysis of 11 RCTs with 13 randomisations involving GO was undertaken. Although GO increased induction deaths (*p* = 0.02), it led to a reduction in resistant disease (*p* = 0.0009); hence, there was no improvement in complete remission. Whilst GO improved relapse-free survival (hazard ratio (HR) = 0.90, 95 % confidence interval (CI) = 0.84–0.98, *p* = 0.01), there was no overall benefit of GO in overall survival (OS) (HR = 0.96, 95 % CI = 0.90–1.02, *p* = 0.2). GO improved OS in patients with favourable cytogenetics, with no evidence of benefit in patients with intermediate or adverse cytogenetics (test for heterogeneity between subtotals *p* = 0.01). GO has a potent clinically detectable anti-leukaemic effect. Further trials to investigate its optimum delivery and identification of patient populations who may benefit are needed.

**Electronic supplementary material:**

The online version of this article (doi:10.1007/s00277-014-2218-6) contains supplementary material, which is available to authorized users.

## Introduction

Despite advances in supportive care, the outcome of patients with acute myeloid leukaemia (AML) remains poor with only about 40 % of adults under 60 years of age achieving long-term survival, and in those over 60 years of age, less than 20 % achieve long-term survival [1]. Immunotherapeutic strategies, utilising antibodies against tumour antigens, have proved highly effective in other haematological malignancies [2] but have yet to become established as standard of care in the management of AML. CD33 is frequently expressed on the surface of AML cells and is rarely expressed outside of the haematological system [3]. Two anti-CD33 antibodies have been evaluated in randomised clinical trials: lintuzumab and gemtuzumab ozogamicin (GO, trade name, Mylotarg). GO is a humanised monoclonal antibody against CD33, conjugated to calicheamicin. Calicheamicin cleaves sequence-specific DNA regions causing double-stranded breaks [4]. GO was reported to improve overall responses dramatically in adults with relapsed AML [5], and as a consequence, GO was given accelerated approval by the US Food and Drug Agency in 2000 but was subsequently voluntarily withdrawn in 2010 because of reports of excessive toxicity [6].

Given the conflicting results of different studies and the potential importance of this drug, the aim of this systematic review and meta-analysis is to assess the totality of the evidence on the effectiveness and optimal delivery of anti-CD33 antibody treatment in AML.

## Methods

### Protocol and eligibility

A study protocol was drawn up prior to the review being undertaken. We sought to include all randomised controlled trials (RCTs) that included any patient with AML, where one arm included anti-CD33 antibody therapy. AML could be in any form: de novo or secondary. Previous treatment was not an excluding factor. There were no age restrictions. We considered all examples of anti-CD33 antibody treatment, with or without conjugation to other molecules, for example toxins. Differing doses and regimens of the treatment did not result in exclusion in this review.

### Search strategy, sources and inclusion criteria

Searches, developed in consultation with an information specialist, were conducted in MEDLINE (1946–2013), Embase (1974–2013) and the Cochrane Library up to December 2013. Major conference proceedings abstracts—American Society of Hematology (ASH) (2004–2013), European Hematology Association (EHA) (2006–2013) and American Society of Clinical Oncology (ASCO) (1996–2013)—were also searched for unpublished trials. Research registers (ClinicalTrials.gov, controlled trials.com, ISRCTN) were searched for ongoing trials. There were no language restrictions. Search strategies are available from the online data supplement.

**Fig. 1:**
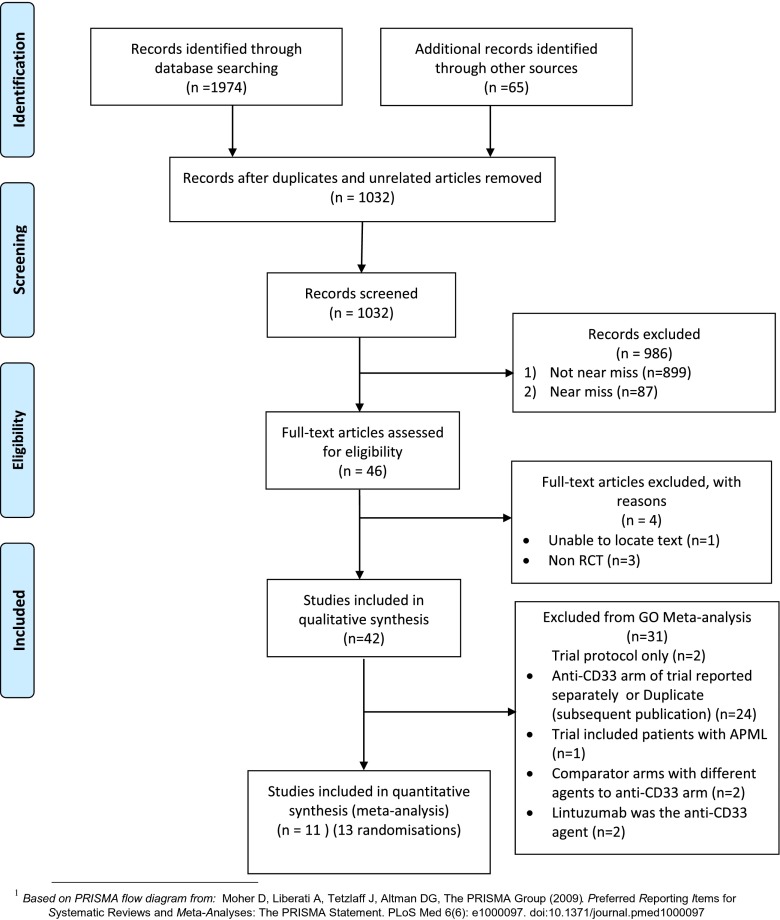
Flow diagram of search strategy (according to PRISMA guidelines)

Studies retrieved from the database searches were reviewed independently by two people (JL and JK or JW), using title and abstract to make inclusion/exclusion decisions. Full paper copies were obtained for further review where there were uncertainties. Disagreement regarding inclusion was resolved by a third member of the team (JW or JK). The selection pathway (Fig. 1) is presented using a PRISMA flow diagram [7].

### Quality assessment and data extraction

Data was extracted by JL and JK or JW independently using a standardised extraction form. The standardised form included details of trial identification details, population characteristics, details of intervention and control treatments, and outcomes. Outcomes were defined by internationally recognised criteria [8] and included overall survival (OS), relapse-free survival (RFS), death in complete remission (DCR), cumulative incidence of relapse (CIR), induction death (ID), resistant disease (RD) and response. Variations in outcome definitions between studies are outlined in the online data supplement. Toxicity data was extracted on a standardised form (JL) and reviewed by JW.

Two reviewers (JL and JK or JW) independently assessed each trial for risk of bias utilising the risk of bias tool developed by the Cochrane Collaboration [9].

### Statistical analysis

Odds ratios (ORs) and 95 % confidence intervals (CIs) were calculated for dichotomous outcomes. Time-to-event data was extracted using standard methods from Tierney [10] and Parmar [11]. Observed minus expected (O − E) number of events and variance were calculated from hazard ratios (HR), CIs, *p* values and survival proportions where available.

Fixed effect meta-analysis methods were used with the *I* ^2^ test for statistical heterogeneity performed. Tests for heterogeneity and trend across predefined subgroups were also performed; these subgroups included age, diagnosis, cytogenetic risk group, *FLT3*-ITD, *NPM1*, CD33 status, stage of treatment, total cumulative dose, with or without other chemotherapy agents and treatment confounding (whether comparator arm had the same dose of concomitant chemotherapy: yes, no). Results are presented as odds ratios with 95 % CIs for dichotomous endpoints and Peto’s odds ratios with 95 % CIs for time-to-event endpoints. Assumptions for outcome data in studies with incomplete reporting, taken after efforts to contact study authors, are stated in the results.

## Results

### Search results

The search yielded 1974 articles (Fig. 1). A search of online clinical trial databases suggested four pending trials. One trial was not due for completion until January 2020 (AML SG09-09; ClinicalTrials.gov identifier: NCT00893399) and would fit our inclusion criteria. Another, NCRI AML17 (ISRCTN55675535), is due to be completed July 2014. The other two were sponsored by pharmaceutical companies; one was terminated early, and the second did not have available data (further supplementary 5).

Two different anti-CD33 therapies were identified in this systematic review: GO, a humanised monoclonal antibody against CD33 conjugated to a toxin, and lintuzumab, a humanised anti-CD33 monoclonal antibody not conjugated to a toxin. Two trials involving lintuzumab were found [12, 13] but only one provided sufficient data for analysis of overall survival; this study [13] did not demonstrate a significant impact of lintuzumab on overall survival (HR = 0.94, 95 % CI = 0.63–1.11); however, this study was small and underpowered. Due to their intrinsic biological differences, these studies were omitted from the main meta-analyses that only involved GO. Thus, 11 trials involving 13 randomisations with 7138 patients entered the meta-analysis of trials involving GO [12–26]. A detailed summary of trials included in this meta-analysis can be found in the supplementary data (further supplementary 4), with a brief summary of each study described in Table 1. We also excluded two small trials from the meta-analysis because they did not include the same concomitant chemotherapy drugs in the GO and the comparator arm [19, 21]. Out of the 11 trials, two had a second independent randomisation at a latter treatment stage [17, 25]. Four studies investigated the use of GO at induction and consolidation phases but with no independent randomisation at the second treatment stage; therefore, these studies contributed to four randomisations only [18, 20, 26, 27]. Three further randomisations involved the use of GO at induction [16, 17, 25], two independent randomisations at consolidation [17, 24] and three at a post-consolidation/maintenance stage [22, 23, 25]. One trial used GO as part of a low-intensity regimen [15]. Up-to-date overall survival and relapse data for the NCRI AML15 trial was used from the AML16 publication [16]. For one study [25], CIR and DCR were calculated from an interim publication [28]; data from this interim analysis was used for the induction randomisation of this study.

**Table 1:** Summary table of trials included in meta-analysis

Trial	Population	Median age	Size	Stage	Dose (mg/m^2^)	Total number of doses	Concomitant chemotherapy	Control chemotherapy
Amadori 2013	APML excluded	67	472	Induction and consolidation	6 (induction), 3 (consolidation)	2 (induction), 2 (consolidation)	MICE (induction), ICE (consolidation)	Same concomitant chemotherapy as in GO arm
Burnett 2011 (induction randomisation)	As above	49	1113	Induction	3	1	DA/ADE/FLAG-IDA	As above
Burnett 2011 (consolidation randomisation)	As above	46	948	Consolidation	3	1	MACE/Ara-C	As above
Burnett 2012 (intensive trial)	AML and high-risk MDS	67	1115	Induction	3	1	DA or d-Clo at induction (2–3 courses), Aza maintenance	As above
Burnett 2012 (low-intensity trial)	AML, high-risk MDS	75	495	Low intensity	Flat dose of 5	4	Low-dose Ara-C 20 mg s/c injection	As above
Castaigne 2012	Primary AML	62	280	Induction and consolidation	3	3 (induction), 2 (consolidation)	DA (1–2 courses at induction); two courses of DA as consolidation	As above
Delaunay 2011 ASH	Intermediate-karyotype AML	50	254	Induction and consolidation	6	2	DA induction and MidAC intensive consolidation	As above
Fernandez 2011	APML excluded	48	270	Consolidation	6	1	None	None
Gamis 2013 ASH	Primary AML	9.9	1070	Induction and consolidation	3	1 (induction), 1 (consolidation)	ADE at induction and mitoxantrone/Ara-C at second consolidation	Same concomitant chemotherapy as in GO arm
Hasle 2012	Standard/high-risk disease in CR1 post-consolidation	Not reported	120	Maintenance	5	2	None	None
Lowenberg 2010	APML excluded	67	232	Maintenance	6	3	None	None
Petersdorf 2013 (induction randomisation)	As above	47	595	Induction	6	1	DA (45 mg/m^2^ daunorubicin)	DA (60 mg/m^2^ daunorubicin)
Petersdorf 2013 (maintenance randomisation)	As above	Not reported	174	Maintenance	5	3	None	None

### Risk of bias

Overall, most publications failed to describe the randomisation and allocation method; without this information, we are unable to assess whether these trials suffered from selection bias. Otherwise, the risk of bias was low for the published trials (supplementary table 1).

Two trials [26, 27] were only available in conference proceedings form which limited assessment of risk of bias and data extraction. Of these, one trial [27] had available data for all outcomes, although assumptions were made for resistant disease and induction death (see further supplementary 3). The other trial [26] only provided data for OS and complete remission (CR) rates. Although data was provided for early deaths, it was unclear whether these deaths were induction related or due to disease. Authors of two unpublished trials [26, 27] were contacted to obtain full trial reports, but additional data was not available for analysis. In comparison, eight of the nine published trials [15–18, 20, 22–25] involving GO provided complete OS data. The exception was the post-consolidation randomisation of one trial [25]. Out of the nine published trials involving GO, one did not provide RFS rates for a separate consolidation randomisation [17] (further supplementary 2).

### The impact of GO on response rates and resistant disease

**Fig. 2:**
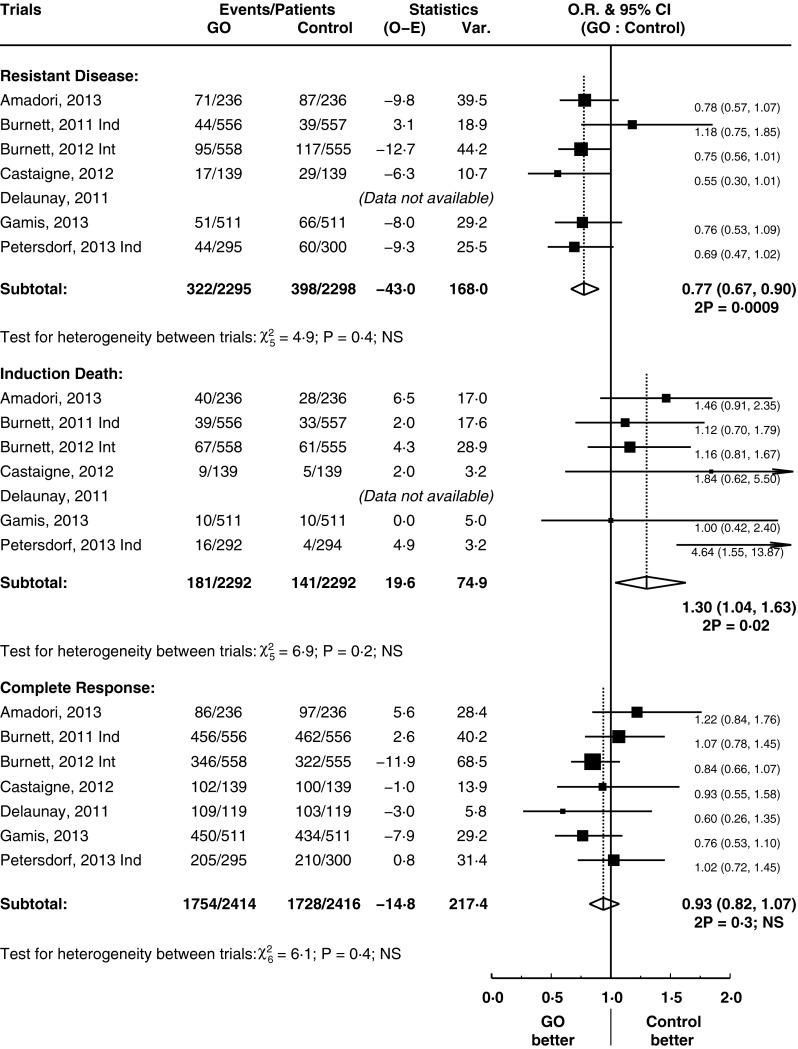
Rates of resistant death, induction death and complete remission. Forest plots (Figs. 2, 3, 4, 5, 6): *black squares* and *horizontal lines* represent estimate and 95 % confidence interval, respectively, for each study. *Open diamond* represents pooled estimates for each subgroup or overall outcome

All induction phase trials had sufficient data for analysis of CR rates. One non-intensive trial was omitted from this analysis because of the different treatment strategy [15]. Overall response (OR) rates (CR plus CR with incomplete count recovery (CRi) or platelet recovery (CRp)) were also calculated; two trial results published as abstract form did not have sufficient data for this analysis [26, 27]. GO does not have a measurable impact on rates of OR, CR nor CRi/p (Fig. 2 and supplementary figure 1). However, the addition of GO to treatment significantly reduced the rate of resistant disease (failure to eliminate disease) by 23 % (HR = 0.77, 95 % CI = 0.67–0.90, *p* = 0.0009) (Fig. 2). However, this was at the expense of a statistically significant increase in induction deaths (HR = 1.30, 95 % CI = 1.04–1.63, *p* = 0.02) (Fig. 2).

### The impact of GO on relapse rates

**Fig. 3:**
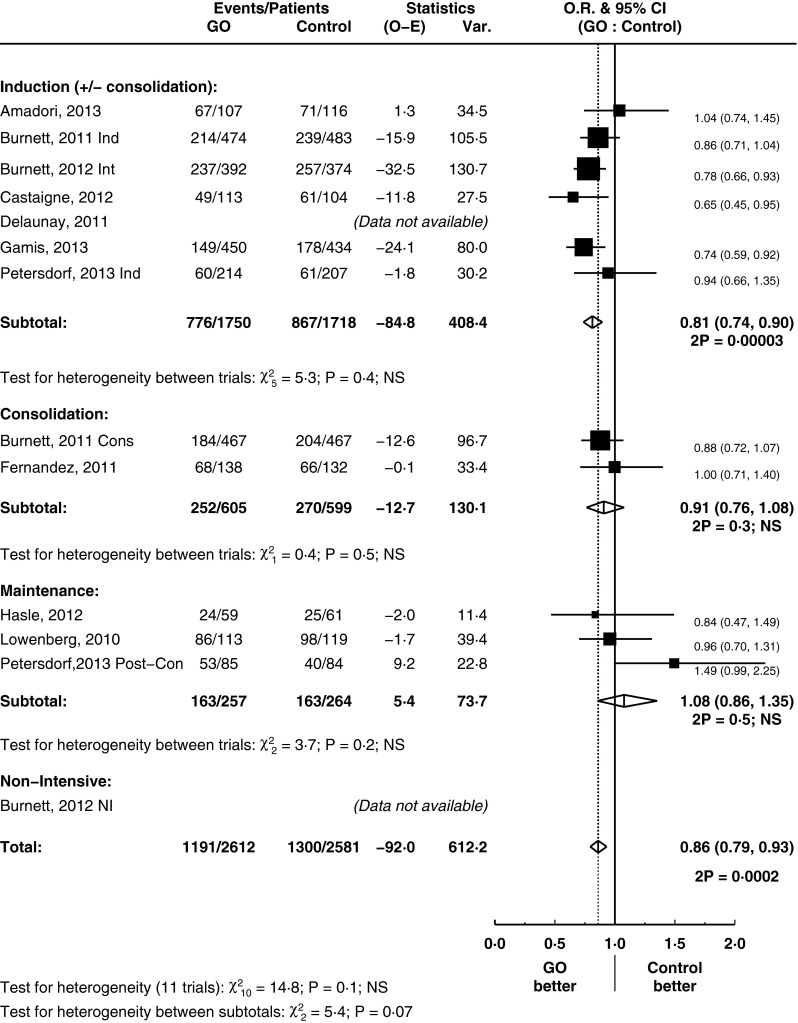
Cumulative incidence of relapse, grouped by treatment stage

**Fig. 4:**
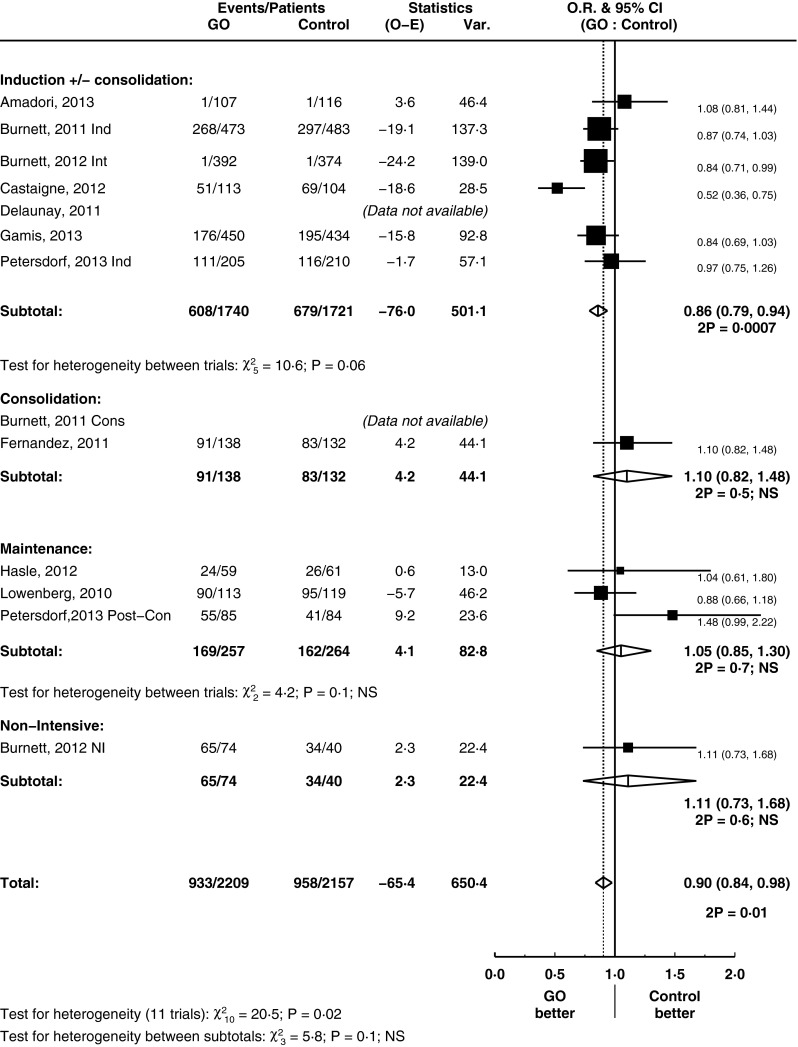
Relapse-free survival, grouped by treatment stage

GO improved CIR by 14 % (HR = 0.86, 95 % CI = 0.79–0.93, *p* = 0.0002) (Fig. 3). This is principally driven by its use in induction treatment where the use of GO reduced the rate of relapse by 19 % (HR = 0.81, 95 % CI = 0.74–0.90, *p* = 0.00003; test for heterogeneity between subtotals *p* = 0.07). GO had no significant influence on the rates of death in CR analysis (HR = 1.11, 95 % CI = 0.91–1.36, *p* = 0.3) (supplementary figure 2). Thus, the improvements in CIR correlated with an overall improvement in RFS in the overall analysis (HR = 0.90, 95 % CI = 0.84–0.98, *p* = 0.01) (Fig. 4).

GO appears to be beneficial in RFS when used as part of remission induction therapy in a subgroup analysis based on treatment stage (HR = 0.86, 95 % CI = 0.79–0.94, *p* = 0.0007). Although the test for heterogeneity between subtotals was borderline (*p* = 0.1), there is a substantial treatment effect seen in induction subgroup. Alongside the significant result for CIR in the setting of remission induction treatment, we explored this subgroup through predefined analyses. Subgroup analysis did not provide clear evidence that effect size of RFS was dependent on the age of patients (median age of trial entrants greater or less than 60), on the cumulative dose (greater or less than 9 mg/m^2^) they received and on whether there was treatment confounding (whether or not the comparative arm had the same dose of accompanying chemotherapy) (test for heterogeneity between subtotals = not significant) (supplementary figures 3–5).

### The effect of GO on overall survival

**Fig. 5:**
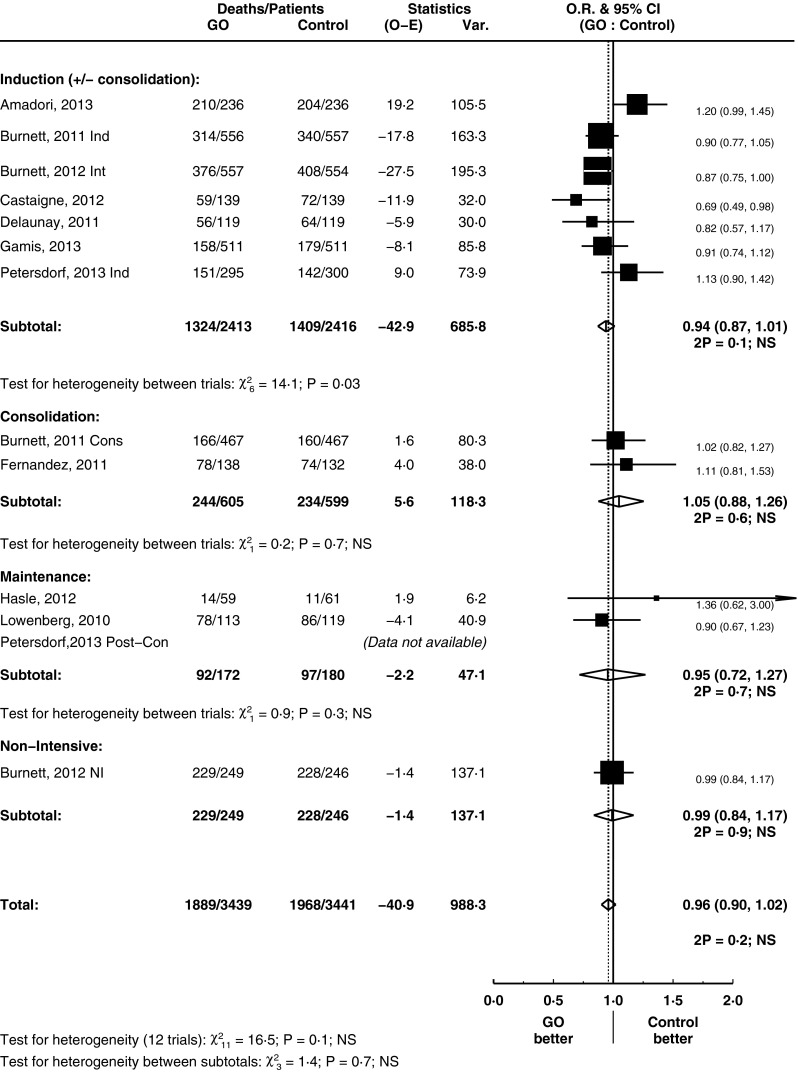
Overall survival, grouped by treatment stage

**Fig. 6:**
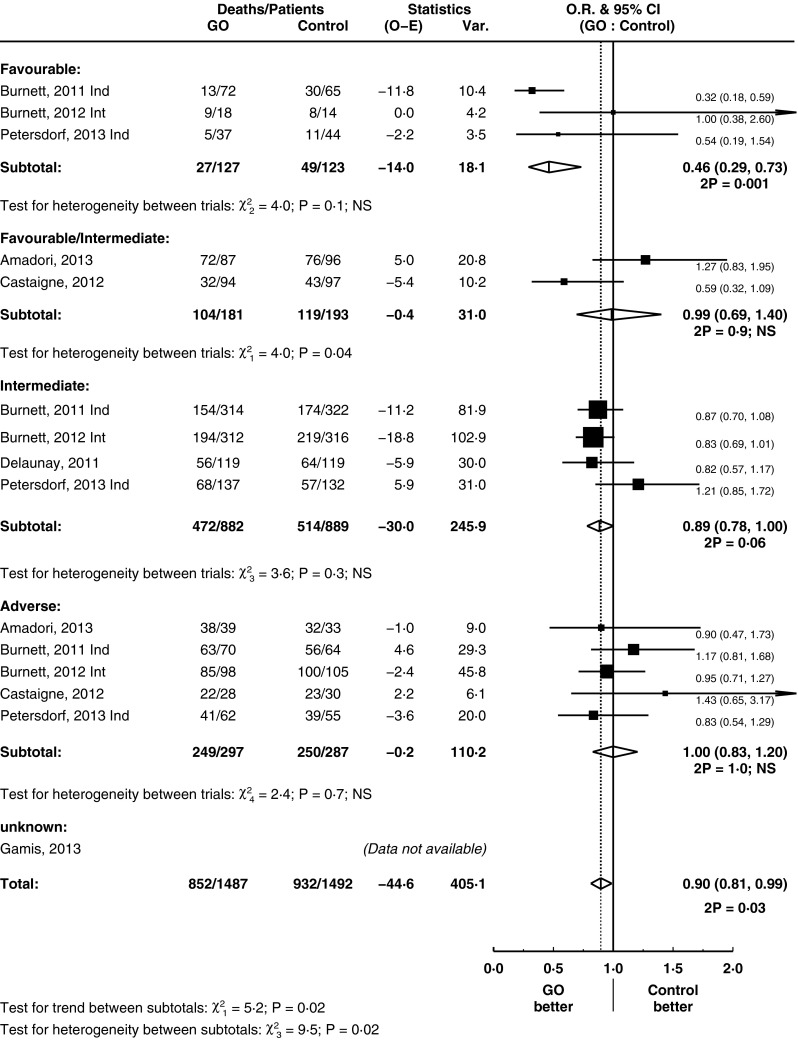
Overall survival, grouped by cytogenetics, for induction remission trials

GO had no significant effect on OS (HR = 0.96, 95 % CI = 0.90–1.02, *p* = 0.2) (Fig. 5), nor was there any evidence for a benefit of GO at any particular treatment stage. However, AML is a heterogeneous condition clinically and by genetic groupings. These analyses were limited by the number of trials involved. However, in one subgroup, there was evidence of benefit from the use of GO in terms of OS: this was in patients with favourable risk cytogenetics (HR = 0.46, 95 % CI = 0.29–0.73, *p* = 0.001) (test for trend between subtotals *p* = 0.02; test for heterogeneity between subtotals *p* = 0.02) (Fig. 6).

GO did not improve OS, in subgroup analyses of induction trials based on age, cumulative dose nor diagnosis (primary vs. secondary AML) (supplementary figures 6–8). Available data for *FLT3*-ITD and NPM1 analysis was limited to only three trials and was unable to detect a significant influence of these subgroups on OS (data not shown). A subgroup analysis based on treatment confounding was performed (supplementary figure 9): there was some evidence of greater benefit for the use of GO in trials without confounding chemotherapy (test for heterogeneity *p* = 0.09). There is no evidence that CD33 positivity influences OS of patients treated with GO (supplementary figure 10).

### Toxicity data for GO

We investigated in detail the increased reports of liver toxicity and incidences of severe veno-occlusive disease (VOD) [29], particularly in the setting of haematopoietic stem cell transplantation [30]. This is displayed in the supplementary data (supplementary table 2), alongside measures of treatment-related fatality. Data was available from four trials using induction treatment (with some coupled with consolidation stage), involving a total of 789 patients [18, 20, 25, 26]. Twelve cases of VOD were reported; however, there was insufficient data available to report the incidence in the control arm. From these trials, there were five fatalities associated with VOD or severe hepatic toxicity when GO was used. Three trials [16, 17, 27] did not specify the number of cases of VOD in the GO arm but declared there were no difference incidence by comparison of study arm. As described above, there was a significant difference in death during induction (Fig. 2 which was extracted from endpoints defined directly as induction deaths [16–18, 20, 25, 27], with one omitted due to data provided only of early deaths [26]).

## Discussion

Our findings show that GO has potent anti-leukaemic effects: there is a significant reduction in treatment-resistant disease by 23 % (*p* = 0.0009), but this is at the expense of an increased induction death rate (*p* = 0.02), leading to no improvement in CR rates. The addition of GO to chemotherapy treatment increases RFS by 10 % (*p* = 0.01) by reducing the rate of disease relapse. The reason for the reduction in relapse is difficult to extrapolate from clinical trials, but the ALFA 0701 group has shown that patients treated with gemtuzumab may have a deeper level of remission, as measured by *NPM1* transcript levels [34].

This systematic review and meta-analysis provides a comprehensive synthesis of the research on the effect of the current anti-CD33 agents on all outcomes, at all treatment stages and age. The meta-analysis of OS and RFS involved 6880 and 4366 patients, respectively. Three meta-analyses [31–33] have recently been published regarding the use of GO with all three concentrating on induction treatment of AML. One study concurs on our finding that GO improves RFS at the expense of early mortality in induction [33] despite the inclusion of a trial [19] that we excluded due to the control treatment having a different concomitant chemotherapy regimen to the GO arm. The second review [32] concurs with our other outcome findings in the induction subgroup analyses (including resistant disease and relapse rates), notably on cytogenetic stratification. In contrast to both reviews, we have included data from more recently published trials [20, 27] which have added data for an additional 1494 patients, including unique data on the use of GO in induction remission in the paediatric setting. The third meta-analysis [31] is an individual patient data (IPD) meta-analysis that included five induction trials [16–18, 25, 26]. This also omits the new data [20, 27] which may be one explanation regarding the differences in conclusions: where in our study we find a statistically significant increase in induction deaths, only 30-day mortality is reported in the IPD meta-analysis from Hills et al., and although there is a trend to favour no GO, this was not statistically significant. Furthermore, whilst they detect a significant improvement in OS in an overall analysis, this is not corroborated by our systematic review and meta-analysis.

The overall effect of GO was seen only in RFS but not in OS. GO improves OS in patients with favourable cytogenetic AML (patients with core-binding factor translocations), with borderline significance for those with intermediate-risk cytogenetics. This suggests that in the treatment of patients with core-binding factor leukaemia, GO should become standard of care. There was insufficient data to comment on the effect of GO on OS in other favourable prognostic groups such as those with NPM1 mutation. This ability to only improve survival in favourable but not poor prognostic AML is in keeping with conventional chemotherapy agents, as opposed to immune-mediated therapies, such as graft-versus-leukaemia effect in allogeneic stem cell transplants which are effective against adverse-risk AML [35]. One possible reason why GO might improve RFS but not OS in the overall analysis is the presence of salvage treatments and subsequent allogeneic haematopoietic stem cell transplantation (HSCT) as a way of consolidating second remissions [36].

In our predefined subgroup analysis of age, we used 60 years of age (based on the median age of trial entrants where available) as a threshold. There is no evidence that the benefit of GO is only restricted to younger patients. This is notable: GO was granted early FDA approval because of success in an older age group [5]. Furthermore, there is a dearth of successful therapeutic options in this cohort of patients with the outlook for elderly patients considerably poorer than for younger patients, even when they are compared in stratified cytogenetic risk groups [37]. Targeted therapies may be better tolerated than conventional chemotherapy. Below 60 years of age, our systematic review resulted in eight randomisations at meta-analysis, only two of which was applicable in the paediatric setting [22, 27]. This is a clinical setting for GO that deserves further attention.

Whilst there is no clear evidence that GO is not effective at other treatment stages, the greatest amount of evidence and the clearest evidence of benefit comes from trials in which GO was used in induction, so we concentrated on these for the subgroup analyses. An analysis of GO based on treatment stage showed it is an effective adjunct in the induction treatment of AML. There is currently no evidence to suggest that it is of benefit in use at any other treatment stages; this suggests optimisation of induction trials warrants the greatest attention. The improvement in RFS is clearly seen when GO was used as part of induction therapy, with improvements of 14 % (*p* = 0.0007) detected driven by improvements in relapse rates of 19 % (*p* = 0.00003).

It is of particular interest to note that the trial [25] which led to withdrawal of GO from the market had a different dose of anthracycline (GO to no GO arm, 45 to 60 mg/m^2^). This stood out on the confounding treatment subgroup analysis, whereas those trials without a confounding treatment had an overall improvement in OS (test for heterogeneity *p* = 0.09). Apart from this potential explanatory factor, there is no clear reason for the heterogeneity observed between induction trials as subgroup analyses did not provide any clear evidence. An initial report of the use of GO in AML delivered a dose of 9 mg/m^2^ [5]. However, a considerable range in terms of both cycle numbers and doses per cycle was seen in subsequent trials. A recent study [18] had suggested their highly fractionated protocol allowed the safe administration of a higher cumulative dose of drug and was part of the reason why they saw a notable benefit for GO in their trial. In contrast, in another study, GO was delivered prior to the use of conventional chemotherapy [20]. This difference in scheduling may be a reason behind the heterogeneity between these two studies seen in our meta-analysis notably in DCR (supplementary figure 2), RFS by treatment stage (Fig. 4) and OS by cytogenetics (Fig. 6). Our study demonstrates that subgroups based on cumulative doses (above and below 9 mg/m^2^) did not show an advantage in OS and RFS for a higher dose. Overall, this suggests that simple increases in the cumulative dose of GO, inevitably associated with higher toxicity, may be unnecessary.

One striking finding was that the benefit of GO was not significantly reliant on CD33 positivity. This analysis was limited by the fact that available published data did not provide outcome data on more detailed CD33 expression stratification, and thus brought together a potentially heterogeneous group. Publications did not detail the size of CD33+/− precursor populations, which is likely to have significant bearing [38] on anti-CD33 treatment efficacy, as this may determine the presence of a residual clone that may escape targeted therapy.

The diverse settings and regimens in which anti-CD33 agents have been employed likely explain the varying results seen with this agent, which has led to questions regarding the overall efficacy of the treatment. There is clear evidence of the anti-leukaemic effects of anti-CD33 therapy with GO in reducing rates of resistant disease and relapse rates. There is no significant effect on overall OS, although there was an OS benefit, based on cytogenetic analysis, in those with favourable core-binding factor translocations. Given the paucity of new agents in the treatment of this condition, this suggests this drug should not be discarded but further trials are warranted to further optimise the delivery of this drug to allow more patients with AML to benefit from this treatment.

## Electronic supplementary material

Below is the link to the electronic supplementary material.

ESM 1 Supplementary figures and tables (supplementary table 1-2 and supplementary figures 1-10) and further supplementary methods and tables (further supplementary 1-5) are available online. (PDF 1.36 mb)

## References

[1] Burnett AK (2012). Treatment of acute myeloid leukemia: are we making progress?. Hematol Am Soc Hematol Educ Program.

[2] Coiffier B, Lepage E, Briére J, Herbrecht R, Tilly H, Bouabdallah R (2002). CHOP chemotherapy plus rituximab compared with CHOP alone in elderly patients with diffuse large-B-cell lymphoma. N Engl J Med.

[3] Tanimoto M, Scheinberg DA, Cordon-Cardo C, Huie D, Clarkson BD, Old LJ (1989). Restricted expression of an early myeloid and monocytic cell surface antigen defined by monoclonal antibody M195. Leukemia.

[4] Zein N, Poncin M, Nilakantan R, Ellestad GA (1989). Calicheamicin gamma 1I and DNA: molecular recognition process responsible for site-specificity. Science.

[5] Sievers EL, Larson RA, Stadtmauer EA, Estey E, Löwenberg B, Dombret H (2001). Efficacy and safety of gemtuzumab ozogamicin in patients with CD33-positive acute myeloid leukemia in first relapse. J Clin Oncol.

[6] US Food and Drug Administration: FDA: Pfizer voluntarily withdraws cancer treatment Mylotarg from US market. http://www.fda.gov/NewsEvents/Newsroom/PressAnnouncements/2010/ucm216448.htm. 2012. Accessed 29th September, 2012

[7] Moher D, Liberati A, Tetzlaff J, Altman D (2009). Preferred reporting items for systematic reviews and meta-analyses: the PRISMA statement. BMJ.

[8] Cheson BD, Bennett JM, Kopecky KJ, Büchner T, Willman CL, Estey EH (2003). Revised recommendations of the international working group for diagnosis, standardization of response criteria, treatment outcomes, and reporting standards for therapeutic trials in acute myeloid leukemia. J Clin Oncol.

[9] Higgins JPT, Green S (editors). Cochrane Handbook for Systematic Reviews of Interventions Version 5.1.0 [updated March 2011]. The Cochrane Collaboration, 2011. Available from www.cochrane-handbook.org

[10] Tierney JF, Stewart LA, Ghersi D, Burdett S, Sydes MR (2007). Practical methods for incorporating summary time-to-event data into meta-analysis. Trials.

[11] Parmar MK, Torri V, Stewart L (1998). Extracting summary statistics to perform meta-analyses of the published literature for survival endpoints. Stat Med.

[12] Feldman EJ, Brandwein J, Stone R, Kalaycio M, Moore J, O’Connor J (2005). Phase III randomized multicenter study of a humanized anti-CD33 monoclonal antibody, lintuzumab, in combination with chemotherapy, versus chemotherapy alone in patients with refractory or first-relapsed acute myeloid leukemia. J Clin Oncol.

[13] Sekeres MA, Lancet JE, Wood BL, Grove LE, Sandalic L, Sievers EL (2013). Randomized, phase IIb study of low-dose cytarabine and lintuzumab versus low-dose cytarabine and placebo in older adults with untreated acute myeloid leukemia. Haematologica.

[14] Burnett AK, Hills RK, Grimwade D, Jovanovic JV, Craig J, McMullin MF (2013). Inclusion of chemotherapy in addition to anthracycline in the treatment of acute promyelocytic leukaemia does not improve outcomes: results of the MRC AML15 trial. Leukemia.

[15] Burnett AK, Hills RK, Hunter AE, Milligan D, Kell WJ, Wheatley K (2013). The addition of gemtuzumab ozogamicin to low-dose Ara-C improves remission rate but does not significantly prolong survival in older patients with acute myeloid leukaemia: results from the LRF AML14 and NCRI AML16 pick-a-winner comparison. Leukemia.

[16] Burnett AK, Russell NH, Hills RK, Kell J, Freeman S, Kjeldsen L (2012). Addition of gemtuzumab ozogamicin to induction chemotherapy improves survival in older patients with acute myeloid leukemia. J Clin Oncol.

[17] Burnett AK, Hills RK, Milligan D, Kjeldsen L, Kell J, Russell NH (2011). Identification of patients with acute myeloblastic leukemia who benefit from the addition of gemtuzumab ozogamicin: results of the MRC AML15 trial. J Clin Oncol.

[18] Castaigne S, Pautas C, Terre C, Raffoux E, Bordessoule D, Bastie JN (2012). Effect of gemtuzumab ozogamicin on survival of adult patients with de-novo acute myeloid leukaemia (ALFA-0701): a randomised, open-label, phase 3 study. Lancet.

[19] Brunnberg U, Mohr M, Noppeney R, Durk HA, Sauerland MC, Muller-Tidow C (2012). Induction therapy of AML with ara-C plus daunorubicin versus ara-C plus gemtuzumab ozogamicin: a randomized phase II trial in elderly patients. Ann Oncol.

[20] Amadori S, Suciu S, Stasi R, Salih HR, Selleslag D, Muus P (2013). Sequential combination of gemtuzumab ozogamicin and standard chemotherapy in older patients with newly diagnosed acute myeloid leukemia: results of a randomized phase III trial by the EORTC and GIMEMA consortium (AML-17). J Clin Oncol.

[21] Litzow MR, Othus M, Cripe LD, Gore SD, Lazarus HM, Lee SJ (2010). Failure of three novel regimens to improve outcome for patients with relapsed or refractory acute myeloid leukaemia: a report from the Eastern Cooperative Oncology Group. Br J Haematol.

[22] Hasle H, Abrahamsson J, Forestier E, Ha SY, Heldrup J, Jahnukainen K (2012). Gemtuzumab ozogamicin as postconsolidation therapy does not prevent relapse in children with AML: results from NOPHO-AML 2004. Blood.

[23] Lowenberg B, Beck J, Graux C, van Putten W, Schouten HC, Verdonck LF (2010). Gemtuzumab ozogamicin as postremission treatment in AML at 60 years of age or more: results of a multicenter phase 3 study. Blood.

[24] Fernandez HF, Sun Z, Litzow MR, Luger SM, Paietta EM, Racevskis J (2011). Autologous transplantation gives encouraging results for young adults with favorable-risk acute myeloid leukemia, but is not improved with gemtuzumab ozogamicin. Blood.

[25] Petersdorf SH, Kopecky KJ, Slovak M, Willman C, Nevill T, Brandwein J (2013). A phase III study of gemtuzumab ozogamicin during induction and post-consolidation therapy in younger patients with acute myeloid leukemia. Blood.

[26] Delaunay J, Recher C, Pigneux A, Witz F, Vey N, Blanchet O et al (2011) Addition of gemtuzumab ozogamycin to chemotherapy improves event-free survival but not overall survival of AML patients with intermediate cytogenetics not eligible for allogeneic transplantation. Results of the GOELAMS AML 2006 IR Study. ASH Annu Meet Abstr 2011, 118: 79

[27] Gamis A, Aplenc R, Alonzo TA, Sung L, Meshinchi S, Gerbing RB (2013). Gemtuzumab ozogamicin (GO) in children with de novo acute myeloid leukemia (AML) improves event-free survival (EFS) by reducing relapse risk results from the randomized phase III Childrens Oncology Group (COG) trial, AAML0531. ASH Annu Meet Abstr.

[28] A phase III study of the addition of gemtuzumab ozogamicin (Mylotarg®) induction therapy versus standard induction with daunomycin and cytosine arabinoside followed by consolidation and subsequent randomization to post-consolidation therapy with gemtuzumab ozogamicin (Mylotarg®) or no additional therapy for patients under age 61 with previously untreated de novo acute myeloid leukemia (AML). http://www.mhlw.go.jp/stf/shingi/2r9852000000vrz2-att/2r9852000000vs34.pdf. Accessed 25th March 2013

[29] Giles FJ, Kantarjian HM, Kornblau SM, Thomas DA, Garcia-Manero G, Waddelow TA (2001). Mylotarg (gemtuzumab ozogamicin) therapy is associated with hepatic venoocclusive disease in patients who have not received stem cell transplantation. Cancer.

[30] Wadleigh M, Richardson PG, Zahrieh D, Lee SJ, Cutler C, Ho V (2003). Prior gemtuzumab ozogamicin exposure significantly increases the risk of veno-occlusive disease in patients who undergo myeloablative allogeneic stem cell transplantation. Blood.

[31] Hills RK, Castaigne S, Appelbaum FR, Delaunay J, Petersdorf S, Othus M (2014). Addition of gemtuzumab ozogamicin to induction chemotherapy in adult patients with acute myeloid leukaemia: a meta-analysis of individual patient data from randomised controlled trials. Lancet Oncol.

[32] Li X, Xu SN, Qin DB, Tan Y, Gong Q, Chen JP (2014). Effect of adding gemtuzumab ozogamicin to induction chemotherapy for newly diagnosed acute myeloid leukemia: a meta-analysis of prospective randomized phase III trials. Ann Oncol.

[33] Kharfan-Dabaja MA, Hamadani M, Reljic T, Pyngolil R, Komrokji RS, Lancet JE (2013). Gemtuzumab ozogamicin for treatment of newly diagnosed acute myeloid leukaemia: a systematic review and meta-analysis. Br J Haematol.

[34] Lambert J, Lambert J, Nibourel O, Pautas C, Hayette S, Cayuela JM (2012). Minimal residual disease assessed by WT1 expression and NPM1 mutations specific RQ-PCR assays identifies patients with distinct outcomes in the ALFA 0701 trial and is decreased by treatment with gemtuzumab ozogamicin. ASH Annu Meet Abstr.

[35] Cornelissen JJ, Breems D, van Putten WL, Gratwohl AA, Passweg JR, Pabst T (2012). Comparative analysis of the value of allogeneic hematopoietic stem-cell transplantation in acute myeloid leukemia with monosomal karyotype versus other cytogenetic risk categories. J Clin Oncol.

[36] Burnett AK, Goldstone A, Hills RK, Milligan D, Prentice A, Yin J (2013). Curability of patients with acute myeloid leukemia who did not undergo transplantation in first remission. J Clin Oncol.

[37] Appelbaum FR, Gundacker H, Head DR, Slovak ML, Willman CL, Godwin JE (2006). Age and acute myeloid leukemia. Blood.

[38] Walter RB, Laszlo GS, Lionberger JM, Pollard JA, Harrington KH, Gudgeon CJ (2014). Heterogeneity of clonal expansion and maturation-linked mutation acquisition in hematopoietic progenitors in human acute myeloid leukemia. Leukemia.

