# Supplementary material for: Mylotarg has potent anti-leukaemic effect: a systematic review and meta-analysis of anti-CD33 antibody treatment in acute myeloid leukaemia
Source: Ann Hematol. 2014 Oct 5;94(3):361–73. doi: 10.1007/s00277-014-2218-6 (PMC4317519; doi:10.1007/s00277-014-2218-6)
Supplement: Supplementary file 1 — Supplementary figures and tables (supplementary table 1-2 and supplementary figures 1-10) and further supplementary methods and tables (further supplementary 1-5) are available online. (PDF 1.36 mb) [file 277_2014_2218_MOESM1_ESM.pdf]

Mylotarg has potent anti-leukaemic effect: a Systematic Review and Meta-analysis of Anti-CD33 Antibody Treatment in Acute Myeloid Leukaemia

Running title

Meta-analysis of Anti-CD33 antibody therapy in AML

Loke J, Khan JN, Wilson JS, Craddock C, Wheatley K

Name: Dr Justin Ching Ting Loke, BM BCh

Address: Centre for Clinical Haematology, Queen Elizabeth Hospital, Birmingham, UK

Name Miss Josephine Naz Khan, MSc

Address: Warwick Medical School, University of Warwick, Coventry, CV4 7AL, UK

Name Ms Jayne S Wilson, MSc

Address: Cancer Research UK Clinical Trials Unit (CRCTU), University of Birmingham, Edgbaston, Birmingham, UK

Name: Prof Charles Craddock, DPhil

Address: Centre for Clinical Haematology, Queen Elizabeth Hospital, Birmingham, UK

Name Prof Keith Wheatley, DPhil (Corresponding author)

Address: Cancer Research UK Clinical Trials Unit (CRCTU), University of Birmingham, Edgbaston, Birmingham, UK

Phone: 0121 415 9119

Fax: 0121 414 3700

E-mail: k.wheatley@bham.ac.uk

Online Supplementary Data

Supplementary Table 1: Summary of risk of bias table of trials included in meta-analysis

Supplementary Table 2: Toxicity of GO treatment

Supplementary figures S1-10: Forest plots, black squares and horizontal lines represent estimate and 95% confidence interval, respectively for each study. Open diamond represent pooled estimates for each subgroup or overall outcome.

S1: Response rates: Overall response (CR and CRi or CRp), Complete Remission (CR) and CR with incomplete count (CRi) or platelet recovery (CRp)

S2: Death in CR

S3: Relapse-free survival, grouped by age (above and below 60 years and paediatrics), for induction trials

S4: Relapse-free survival, grouped by dose (above and below 9mg/m^2), for induction trials

S5: Relapse-free survival, grouped by presence of treatment confounder (presence or absence), for induction trials

S6: Overall survival, grouped by age (above and below 60 years and paediatrics), for induction trials

S7: Overall survival, grouped by dose (above and below 9mg/m^2), for induction trials

S8: Overall survival, grouped by diagnosis (primary versus secondary AML), for induction trials

S9: Overall survival, grouped by presence of treatment confounder (presence or absence), for induction trials

S10: Overall survival, grouped by CD33 positivity (positive versus negative), for induction trials

Supplementary Table 1 Summary of risk of bias table of trials included in meta-analysis

	**Selection**	**Performance**	**Attrition**	**Reporting**	**Other**	**Overall risk of Bias**
Amadori 2013	Low	Low	Low	Low	Low	Low
Brunnberg 2012	Unclear	Low	Low	Low	Low	Low
Burnett 2011	Low	Low	Low	Low	Low	Low
Burnett 2011 (consolidation randomisation)	Low	Low	Low	Low	Low	Low
Burnett 2012 (intensive trial)	Low	Low	Low	Low	Low	Low
Burnett 2012 (low intensity trial)	Low	Low	Low	Low	Low	Low
Castaigne 2012	Low	Low	Low	Low	Low	Low
Delaunay 2011 ASH	Unclear	Low	Unclear	Unclear	Unclear	n/a
Fernandez 2011	Unclear	Low	Low	Low	Low	Low
Gamis 2013 ASH	Unclear	Low	Unclear	Unclear	Low	n/a
Hasle 2012	Unclear	Low	Low	Low	Low	Low
Litzow 2010	Unclear	Low	Low	Low	Unclear	Low
Lowenberg 2010	Unclear	Low	Low	Low	Low	Low
Petersdorf 2013 (induction randomisation)	Unclear	Low	Low	Low	Low	Low
Petersdorf 2013 (maintenance randmisation)	Unclear	Low	Low	Low	Low	Low

Supplementary Table 2

Toxicity of GO treatment

Trial	Intensive vs non intensive	Median Age	Cohort Size	Treatment Stage	Hepatic Toxicity	VOD in GO arm	Treatment related fatality GO (%)	Treatment related fatality in control (%)	Definition of Treatment related Fatality	Definition of Hepatic Toxicity
Amadori 2013	Intensive	67	472	Induction and consolidation	34 (15%) in GO arm. 23 (10%) in no GO arm.	Two fatalities related to VOD at induction one fatality at consolidation with VOD.	40 (17%)	27 (12%)	Induction death as a result of treatment related toxicities	≥3 Grade National Cancer Institute (NCI) Common Toxicity Criteria (CTC) version 3.0 (http://ctep.cancer.gov/reporting/ctc.html)
Burnett 2011	Intensive	49	1119	Induction and Consolidation (Separate randomisation)	"No excess liver toxicity seen"	-	39 (7%)	33 (6%)	Induction death (treatment and/or hypoplasia related fatality within 30 days.) No separate data for induction and consolidation phases.	Not specified.
Burnett 2012 JCO (Intensive trial)	Intensive	67	1115	Induction	7% GO, 6% No GO	-	67 (12%)	61 (11%)	Induction death	NCI CTC V3 Grade3-4 Bilirubin rise
Burnett 2012 Leukaemia (low intensity trial)	Non-intensive	75	495	Low intensity	Course 1: 4% GO, 3% No GO. Course 2: Both arms 15.	None observed	18%	16%	30 day mortality.	NCI CTC V3 Grade3-4 Bilirubin rise
Castaigne 2012	Intensive	62	280	Induction and Consolidation	18 (13%) in GO arm vs. 9(6%) in no GO arm RR 0.5 (95% CI 0.24, 1.05) P = 0.10	3 in GO arm. 2 subsequently died.	9 (6%)	5 (4%)	Induction death.	NCI CTC V3 Grade3-4 liver toxicity.
Delaunay 2011	Intensive	50	254	Induction and Consolidation	27 (23%) in GO arm, 15 (13%) in no GO arm P = 0.031	4	12 (10%)	5 (4.5%)	Abstract only. Grade3-4 liver toxicity. Early deaths.	Not defined- abstract available only.

Fernandez 2011	Intensive	48	270	Consolidation	No fatalities due to liver dysfunction.	6 in GO arm after auto HSCT	-	-	Overall trial TRM for auto HSCT 2.3%.	Unclear
Gamis 2013	Intensive	9.9 (GO arm); 9.5 (No GO arm)	1022	Induction and consolidation		VOD observed in 3% (severe in 0.6%)-no differences seen by study arm	2% in induction, 5% overall with no difference by study arm	-	"Treatment mortality"	Not defined- abstract available only.
Hasle 2012	Intensive	Paediatric	120	Maintenance	0 in both arms.	None observed	None observed	None observed	Not applicable	WHO Grade 3-4 liver toxicity. From patients who received 2 GO courses.
Lowenberg 2010	Intensive	67	232	Maintenance	19 (17%) in GO arm, no data for control.	1 (likely related to separate post relapse therapy)	7 (6%)	2 (2%)	Control arm- no treatment. Deaths in CR. One death due to treatment related acute liver failure.	CTC grade 2-4
Petersdorf 2013	Intensive	47 (induction)	595 (induction) 169 (maintenance	Induction and maintenance (separate randomisation)	1 hepatic failure related death in GO arm at induction, no fatal related incidence in control arm at induction. 1 Grade 4 at maintenance in GO arm.	3 at induction (from online data summary, 2010) [1]	16 (5%)	4 (1%)	No fatality at maintenance.	NCI CTC V3

Supplementary figures S1-10: Forest plots, black squares and horizontal lines represent estimate and 95% confidence interval, respectively for each study. Open diamond represent pooled estimates for each subgroup or overall outcome.

Supplementary figure 1: Overall response (CR and CRi or CRp), Complete Remission (CR) and CR with incomplete count (CRi) or platelet recovery (CRp)

Response (excl trials with no data)

Trials	Events/Patients GO	Events/Patients Control	Statistics (O–E)	Statistics Var.	O.R. & 95% CI (GO : Control)
**OR:**					
Amadori, 2013	107/238	116/236	4·5	29·8	1.16 (0.81, 1.66)
Burnett, 2011 Ind	473/556	484/556	5·1	33·6	1.16 (0.83, 1.63)
Burnett, 2012 Int	394/558	376/555	−8·3	59·5	0.87 (0.67, 1.12)
Castaigne, 2012	113/139	104/139	−4·5	11·9	0.69 (0.39, 1.21)
Petersdorf, 2013 Ind	224/295	222/300	−2·9	28·0	0.90 (0.62, 1.31)
**Subtotal:**	**1311/1786**	**1302/1786**	**−6·1**	**162·8**	**0.96 (0.83, 1.12) 2P = 0·6**
Test for heterogeneity between trials: χ^2_4 = 4·4; P = 0·4					
**CR:**					
Amadori, 2013	86/236	97/236	5·6	28·4	1.22 (0.84, 1.76)
Burnett, 2011 Ind	456/556	462/556	2·6	40·2	1.07 (0.78, 1.45)
Burnett, 2012 Int	346/558	322/555	−11·9	68·5	0.84 (0.66, 1.06)
Castaigne, 2012	102/139	100/139	−1·0	13·9	0.93 (0.55, 1.58)
Delaunay, 2011	109/119	103/119	−3·0	5·8	0.60 (0.26, 1.35)
Gamis, 2013	450/511	434/511	−7·9	29·2	0.76 (0.53, 1.10)
Petersdorf, 2013 Ind	205/295	210/300	0·8	31·4	1.02 (0.72, 1.45)
**Subtotal:**	**1754/2414**	**1728/2416**	**−14·8**	**217·4**	**0.93 (0.82, 1.07) 2P = 0·3**
Test for heterogeneity between trials: χ^2_6 = 6·1; P = 0·4					
**CRi/CRp:**					
Amadori, 2013	21/236	19/236	−1·0	9·1	0.89 (0.47, 1.71)
Burnett, 2011 Ind	17/556	22/556	2·5	9·4	1.30 (0.69, 2.47)
Burnett, 2012 Int	50/558	56/555	3·1	24·0	1.14 (0.76, 1.70)
Castaigne, 2012	11/139	4/139	−3·5	3·6	0.37 (0.13, 1.06)
Petersdorf, 2013 Ind	19/295	12/300	−3·6	7·4	0.61 (0.30, 1.26)
**Subtotal:**	**118/1784**	**113/1786**	**−2·5**	**53·4**	**0.95 (0.73, 1.25) 2P = 0·7**
Test for heterogeneity between trials: χ^2_4 = 6·3; P = 0·2					

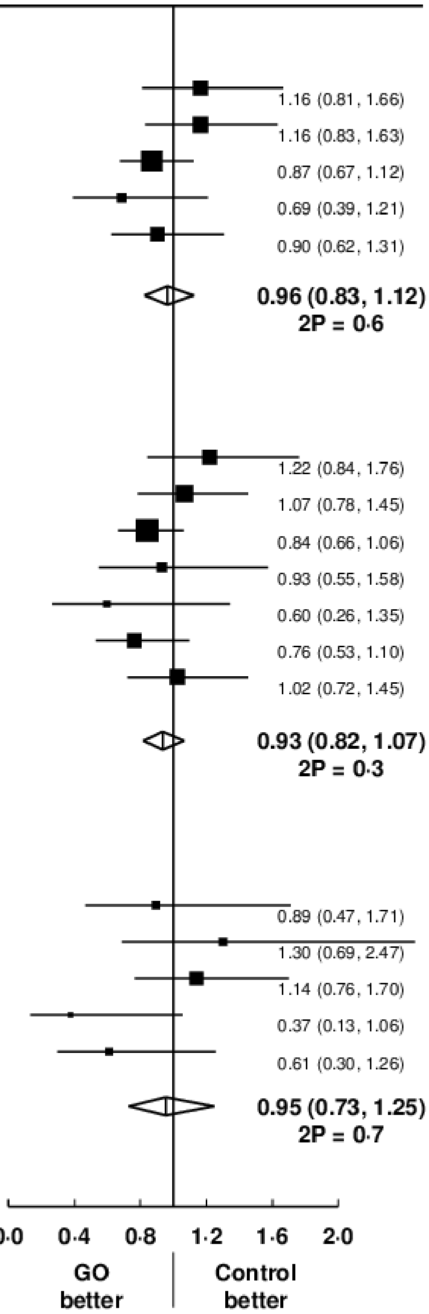

Supplementary figure 2**:** Death in CR

DCR by Treatment Stage

	Events/Patients GO	Events/Patients Control	Statistics (O–E)	Statistics Var.
**Induction (+/– consolidation):**				
Amadori, 2013	15/107	16/116	0·1	7·8
Burnett, 2011 Ind	62/473	69/483	–4·7	33·5
Burnett, 2012 Int	47/392	30/374	8·0	19·3
Castaigne, 2012	2/113	8/104	–2·6	1·6
Delaunay, 2011		*(Data not available)*		
Gamis, 2013	27/450	17/434	4·7	11·0
Petersdorf, 2013 Ind	20/214	19/207	0·2	9·7
**Subtotal:**	**173/1749**	**159/1718**	**5·8**	**82·9**
Test for heterogeneity between trials: χ^2_5 = 9·9; P = 0·08				
**Consolidation:**				
Burnett, 2011 Cons		*(Data not available)*		
Fernandez, 2011	22/138	18/132	1·7	9·9
**Subtotal:**	**22/138**	**18/132**	**1·7**	**9·9**
**Maintenance:**				
Hasle, 2012	0/59	1/61		
Lowenberg, 2010	7/113	2/119	2·2	1·6
Petersdorf,2013 Post–Con	2/85	1/84	0·5	0·8
**Subtotal:**	**9/257**	**4/264**	**2·7**	**2·3**
Test for heterogeneity between trials: χ^2_1 = 0·3; P = 0·6; NS				
**Total:**	**204/2144**	**181/2114**	**10·1**	**95·1**

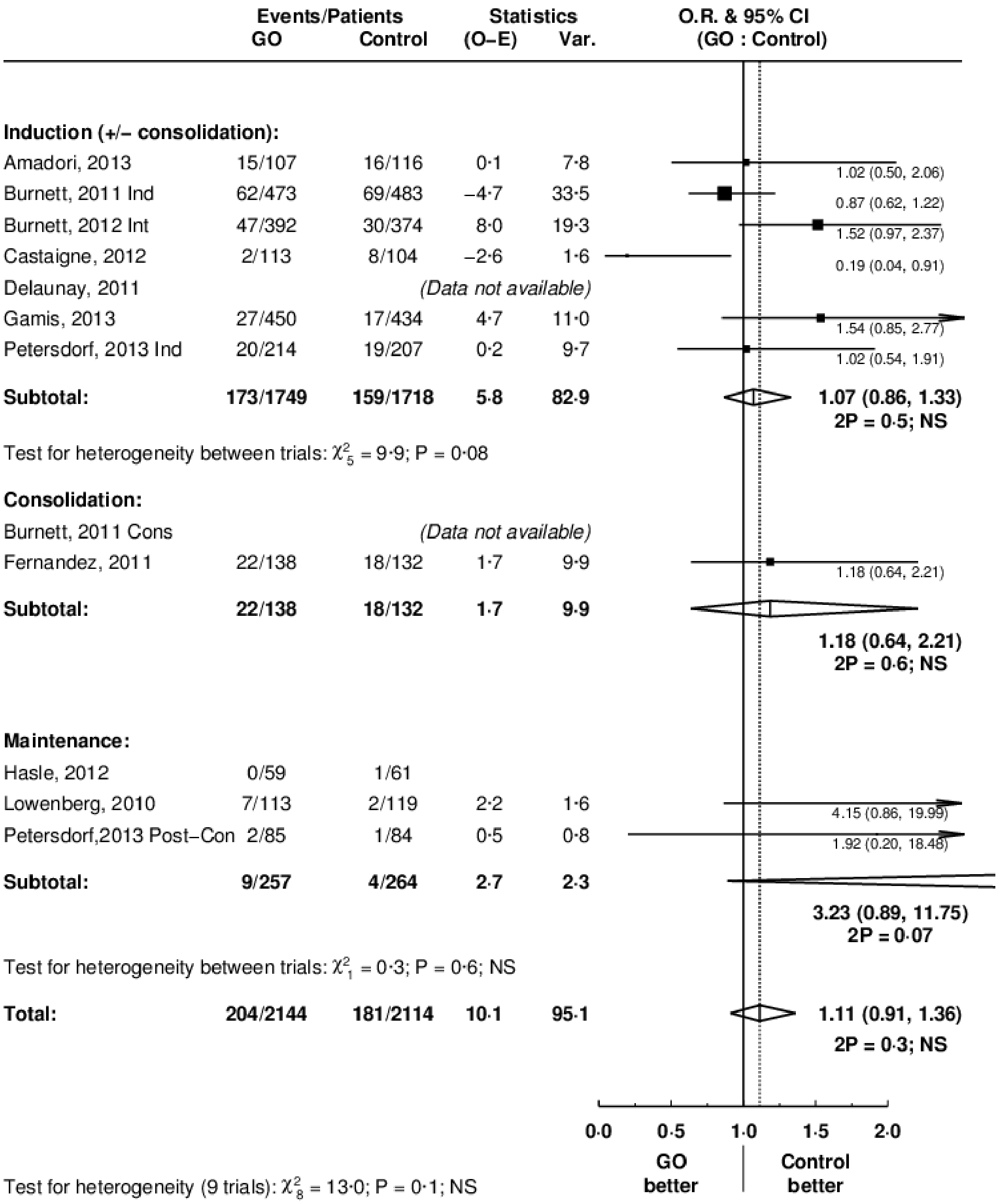

Test for heterogeneity (9 trials): χ^2_8 = 13·0; P = 0·1; NS

Test for heterogeneity between subtotals: χ^2_2 = 2·8; P = 0·2; NS

Supplementary figure 3**:** Relapse-free survival, grouped by age (above and below 60 years and paediatrics), for induction trials

RFS by Age – induction trials

	Events/Patients GO	Events/Patients Control	Statistics (O–E)	Statistics Var.	O.R. & 95% CI (GO : Control)
**Paediatrics:**					
Gamis, 2013	176/450	196/434	−15·8	92·8	0.84 (0.69, 1.03)
**Subtotal:**	**176/450**	**196/434**	**−15·8**	**92·8**	**0.84 (0.69, 1.03)**
**<60 years:**					
Burnett, 2011 Ind	268/473	297/483	−19·1	137·3	0.87 (0.74, 1.03)
Delaunay, 2011		*(Data not available)*			
Petersdorf, 2013 Ind	111/205	116/210	−1·7	57·1	0.97 (0.75, 1.26)
**Subtotal:**	**379/678**	**413/693**	**−20·8**	**194·4**	**0.90 (0.78, 1.03) 2P = 0·1; NS**
Test for heterogeneity between trials: $\chi^2_1 = 0·5$; P = 0·5; NS					
**> 60 years:**					
Amadori, 2013	1/107	1/116	3·6	46·4	1.08 (0.81, 1.44)
Burnett, 2012 Int	1/392	1/374	−24·2	139·0	0.84 (0.71, 0.99)
Castaigne, 2012	51/113	69/104	−18·6	28·5	0.52 (0.36, 0.75)
**Subtotal:**	**53/612**	**71/594**	**−39·3**	**214·0**	**0.83 (0.73, 0.95) 2P = 0·007**
Test for heterogeneity between trials: $\chi^2_2 = 9·5$; P = 0·009					
**Total:**	**608/1740**	**680/1721**	**−76·0**	**501·1**	**0.86 (0.79, 0.94) 2P = 0·0007**

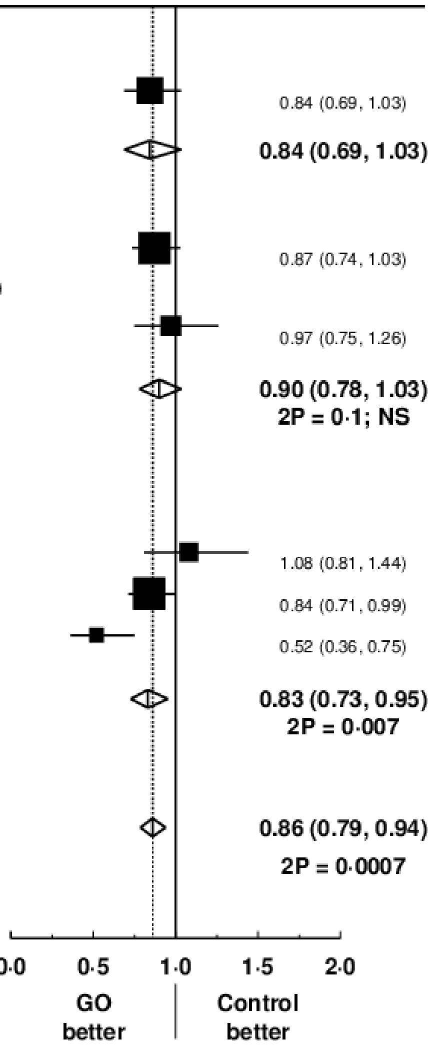

Test for trend between subtotals: $\chi^2_1 = 0·1$; P = 0·8; NS
Test for heterogeneity between subtotals: $\chi^2_2 = 0·6$; P = 0·7; NS

Supplementary figure 4: Relapse-free survival, grouped by dose (above and below 9mg/m2), for induction trials

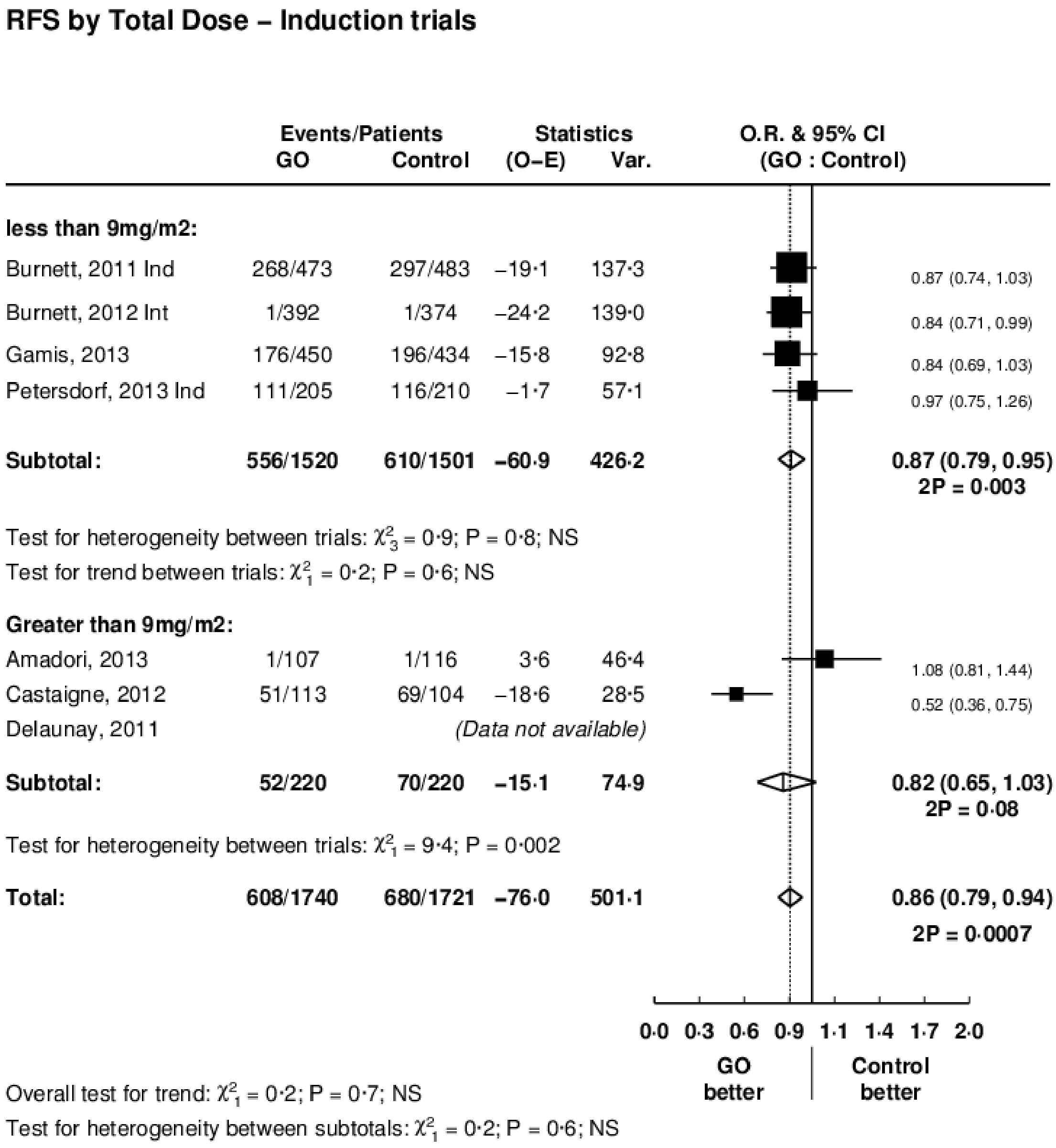

Supplementary figure 5: Relapse-free survival, grouped by presence of treatment confounder (presence or absence), for induction trials

RFS by treatment confounding – induction trials

	Events/Patients GO	Events/Patients Control	Statistics (O–E)	Statistics Var.	O.R. & 95% CI (GO : Control)
**Yes:**					
Petersdorf, 2013 Ind	111/205	116/210	−1·7	57·1	0.97 (0.75, 1.26)
**Subtotal:**	**111/205**	**116/210**	**−1·7**	**57·1**	**0.97 (0.75, 1.26)** **2P = 0·8; NS**
**No:**					
Amadori, 2013	1/107	1/116	3·6	46·4	1.08 (0.81, 1.44)
Burnett, 2011 Ind	268/473	297/483	−19·1	137·3	0.87 (0.74, 1.03)
Burnett, 2012 Int	1/392	1/374	−24·2	139·0	0.84 (0.71, 0.99)
Castaigne, 2012	51/113	69/104	−18·6	28·5	0.52 (0.36, 0.75)
Delaunay, 2011	275/450	239/434	−24·1	80·0	0.74 (0.59, 0.92)
Gamis, 2013	176/450	196/434	−15·8	92·8	0.84 (0.69, 1.03)
**Subtotal:**	**772/1985**	**803/1945**	**−98·4**	**524·1**	**0.83 (0.76, 0.90)** **2P = 0·00002**
Test for heterogeneity between trials: $\chi^2_5 = 10{\cdot}9$; P = 0·05					
**Total:**	**883/2190**	**919/2155**	**−100·1**	**581·2**	**0.84 (0.78, 0.91)** **2P = 0·00003**

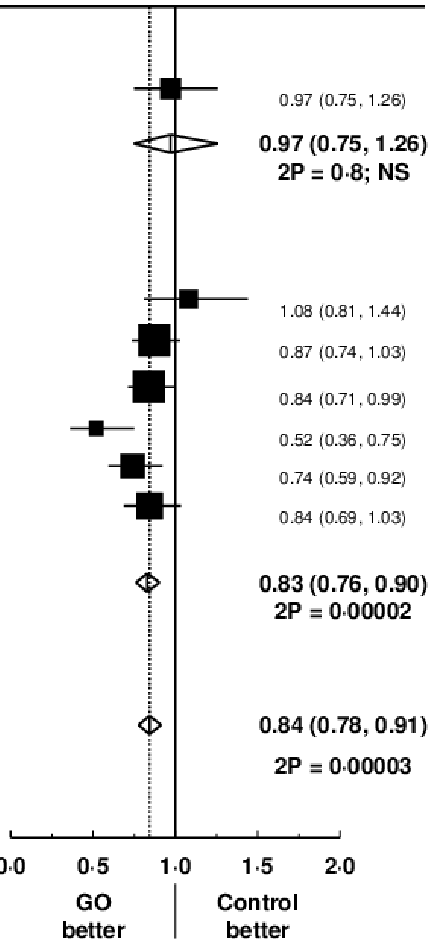

Test for heterogeneity between subtotals: $\chi^2_1 = 1{\cdot}3$; P = 0·3; NS

Supplementary figure 6: Overall survival, grouped by age (above and below 60 years and paediatrics), for induction trials

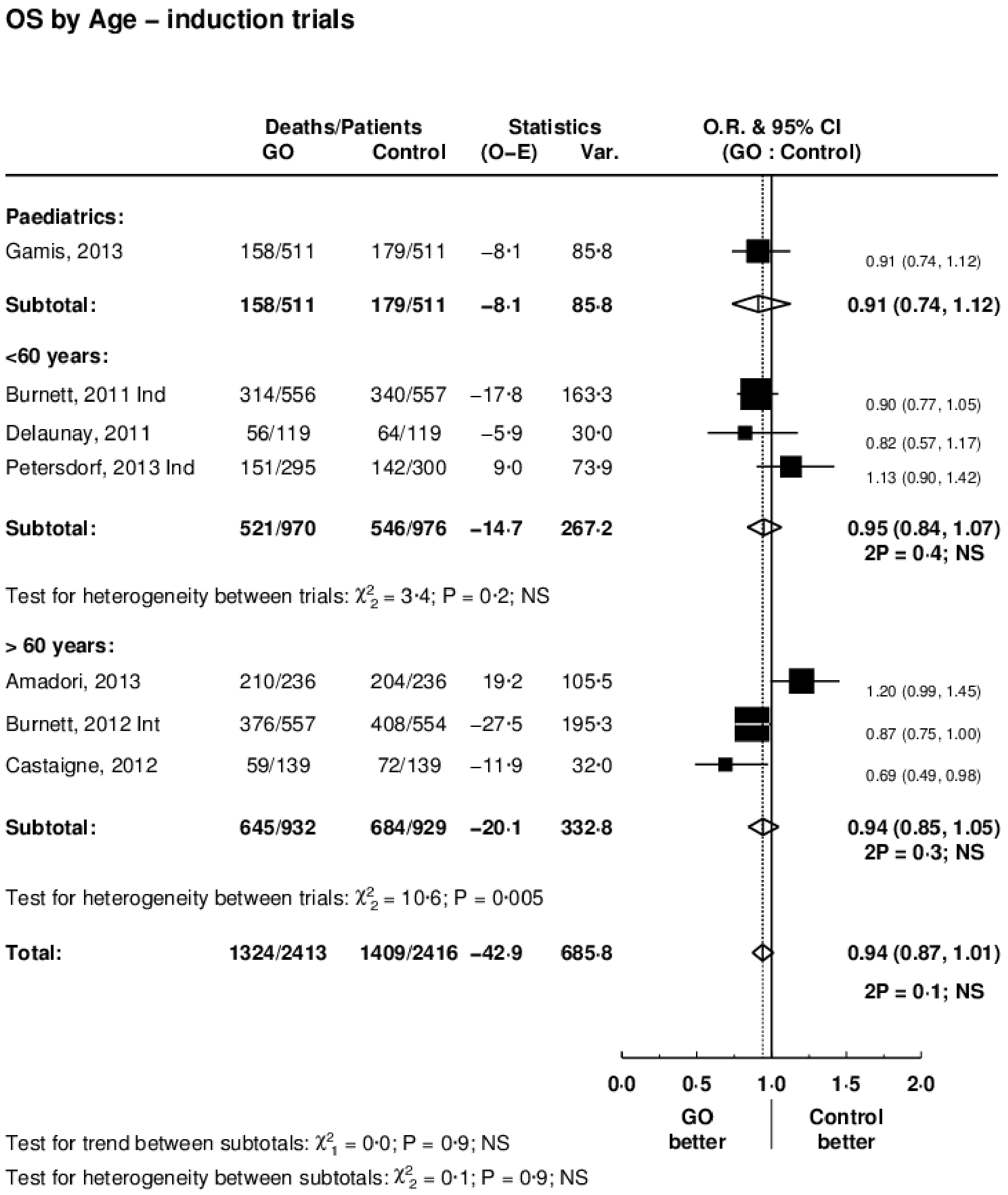

Supplementary figure 7: Overall survival, grouped by dose (above and below 9mg/m2), for induction trials

OS by Total Dose – Induction trials

	Deaths/Patients GO	Deaths/Patients Control	Statistics (O–E)	Statistics Var.	O.R. & 95% CI (GO : Control)
**less than 9mg/m2:**					
Burnett, 2011 Ind	314/556	340/557	–17·8	163·3	0.90 (0.77, 1.05)
Burnett, 2012 Int	376/557	408/554	–27·5	195·3	0.87 (0.75, 1.00)
Petersdorf, 2013 Ind	151/295	142/300	9·0	73·9	1.13 (0.90, 1.42)
Gamis, 2013	158/511	179/511	–8·1	85·8	0.91 (0.74, 1.12)
**Subtotal:**	**999/1919**	**1069/1922**	**–44·4**	**518·2**	**0.92 (0.84, 1.00) 2P = 0·05**
**Greater than 9mg/m2:**					
Amadori, 2013	210/236	204/236	19·2	105·5	1.20 (0.99, 1.45)
Castaigne, 2012	59/139	72/139	–11·9	32·0	0.69 (0.49, 0.98)
Delaunay, 2011	56/119	64/119	–5·9	30·0	0.82 (0.57, 1.17)
**Subtotal:**	**325/494**	**340/494**	**1·5**	**167·5**	**1.01 (0.87, 1.17) 2P = 0·9; NS**
**Total:**	**1324/2413**	**1409/2416**	**–42·9**	**685·8**	**0.94 (0.87, 1.01) 2P = 0·1; NS**

Less than 9mg/m2 — Test for heterogeneity between trials: $\chi^2_3 = 3{\cdot}9$; P = 0·3; NS

Test for trend between trials: $\chi^2_1 = 0{\cdot}5$; P = 0·5; NS

Greater than 9mg/m2 — Test for heterogeneity between trials: $\chi^2_2 = 9{\cdot}1$; P = 0·01

Test for trend between trials: $\chi^2_1 = 5{\cdot}9$; P = 0·02

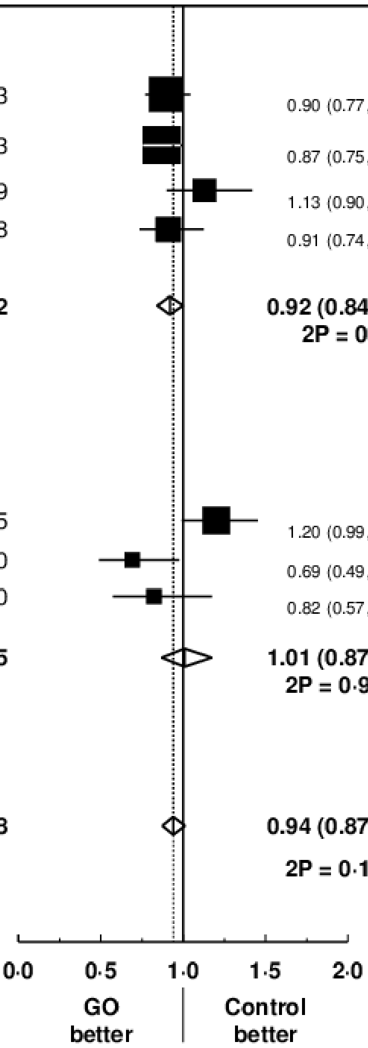

Overall test for trend: $\chi^2_1 = 0{\cdot}5$; P = 0·5; NS

Test for heterogeneity between subtotals: $\chi^2_1 = 1{\cdot}1$; P = 0·3; NS

Supplementary figure 8: Overall survival, grouped by diagnosis (primary versus secondary AML), for induction trials

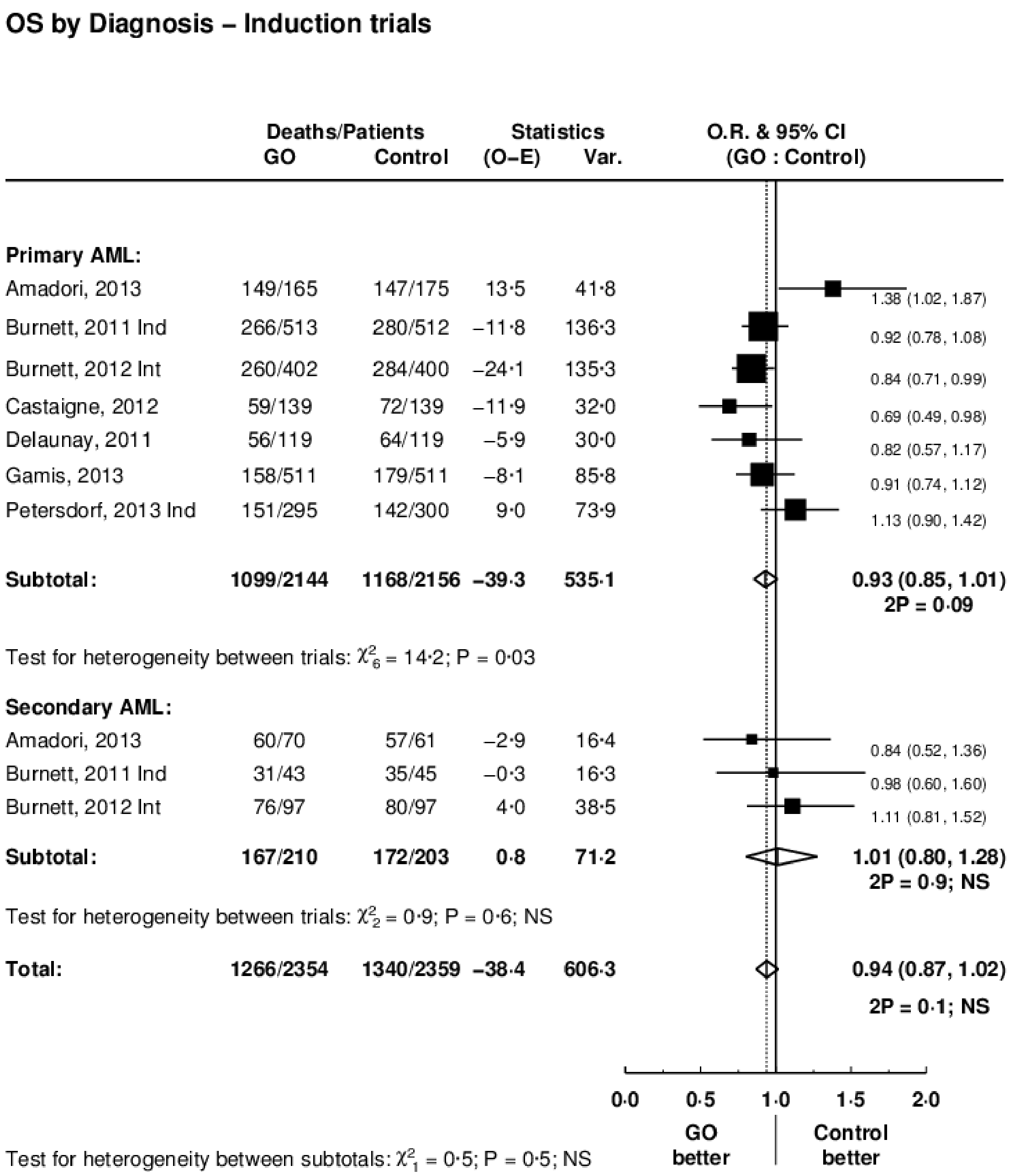

Supplementary figure 9: Overall survival, grouped by presence of treatment confounder (presence or absence), for induction trials

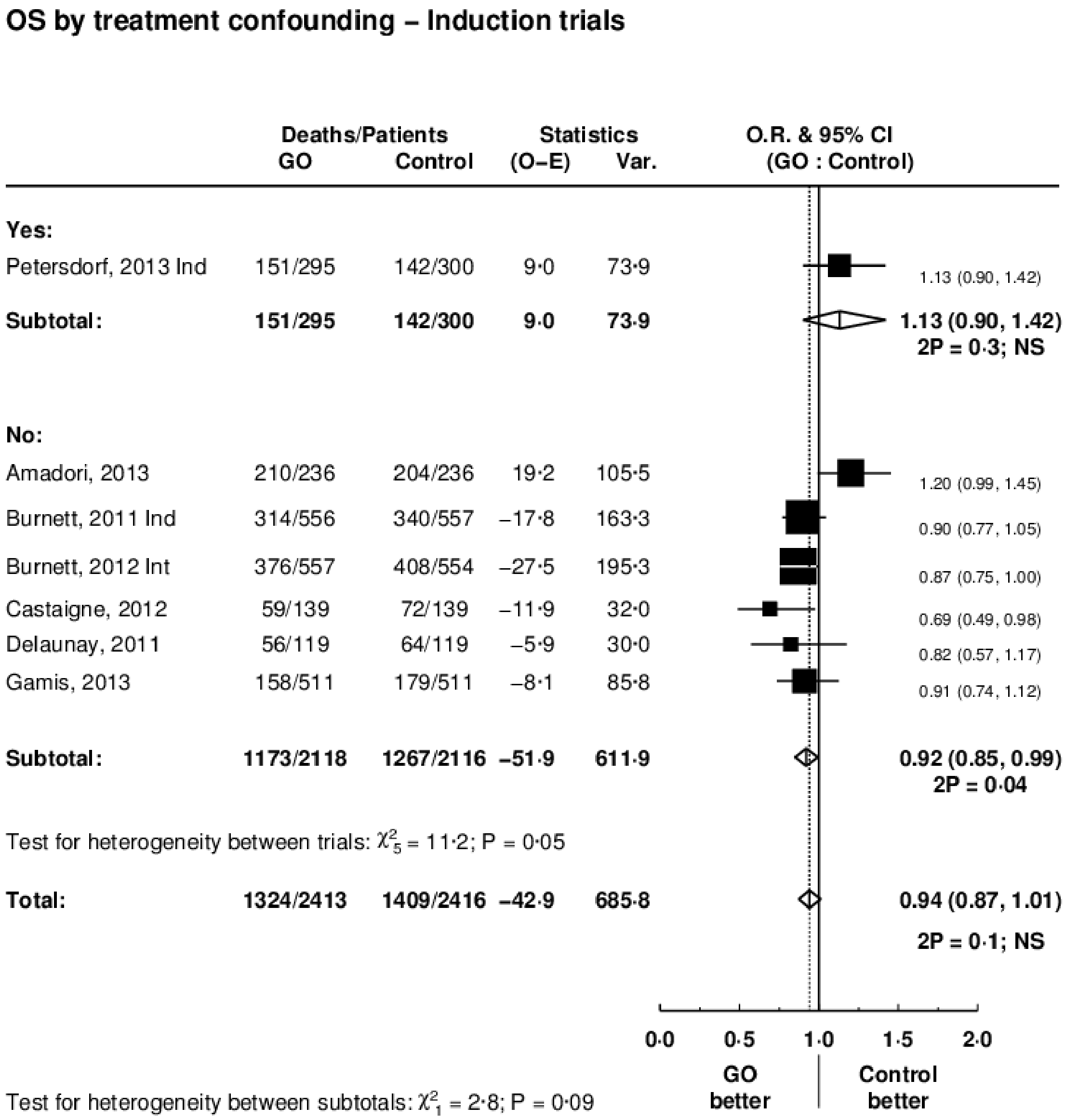

Supplementary figure 10: Overall survival, grouped by CD33 positivity (positive versus negative), for induction trials

Overall Survival by CD33 – induction trials

Trials	Deaths/Patients GO	Deaths/Patients Control	Statistics (O–E)	Statistics Var.	O.R. & 95% CI (GO : Control)
**CD33 +ve:**					
Amadori, 2013	185/206	177/205	10·3	90·5	1.12 (0.91, 1.38)
Burnett, 2011 Ind	210/418	234/416	–17·1	110·8	0.86 (0.71, 1.03)
Burnett, 2012 Int	254/390	274/382	–21·1	131·2	0.85 (0.72, 1.01)
Delaunay, 2011	56/119	64/119	–5·9	30·0	0.82 (0.57, 1.17)
**Subtotal:**	**705/1133**	**749/1122**	**–33·8**	**362·5**	**0.91 (0.82, 1.01) 2P = 0·08**
Test for heterogeneity between trials: $\chi^2_3 = 5·2$; P = 0·2; NS					
**CD33 –ve:**					
Amadori, 2013	23/28	25/29	–1·4	12·0	0.89 (0.50, 1.56)
Burnett, 2011 Ind	26/43	22/33	–0·8	11·8	0.93 (0.53, 1.65)
Burnett, 2012 Int	42/55	42/56	–0·2	20·7	0.99 (0.64, 1.52)
**Subtotal:**	**91/126**	**89/118**	**–2·4**	**44·5**	**0.95 (0.71, 1.27) 2P = 0·7; NS**
Test for heterogeneity between trials: $\chi^2_2 = 0·1$; P = 1·0; NS					
**unknown:**					
Castaigne, 2012		*(Data not available)*			
Gamis, 2013		*(Data not available)*			
Petersdorf, 2013 Ind		*(Data not available)*			
**Total:**	**796/1259**	**838/1240**	**–36·2**	**407·0**	**0.91 (0.83, 1.01) 2P = 0·07**

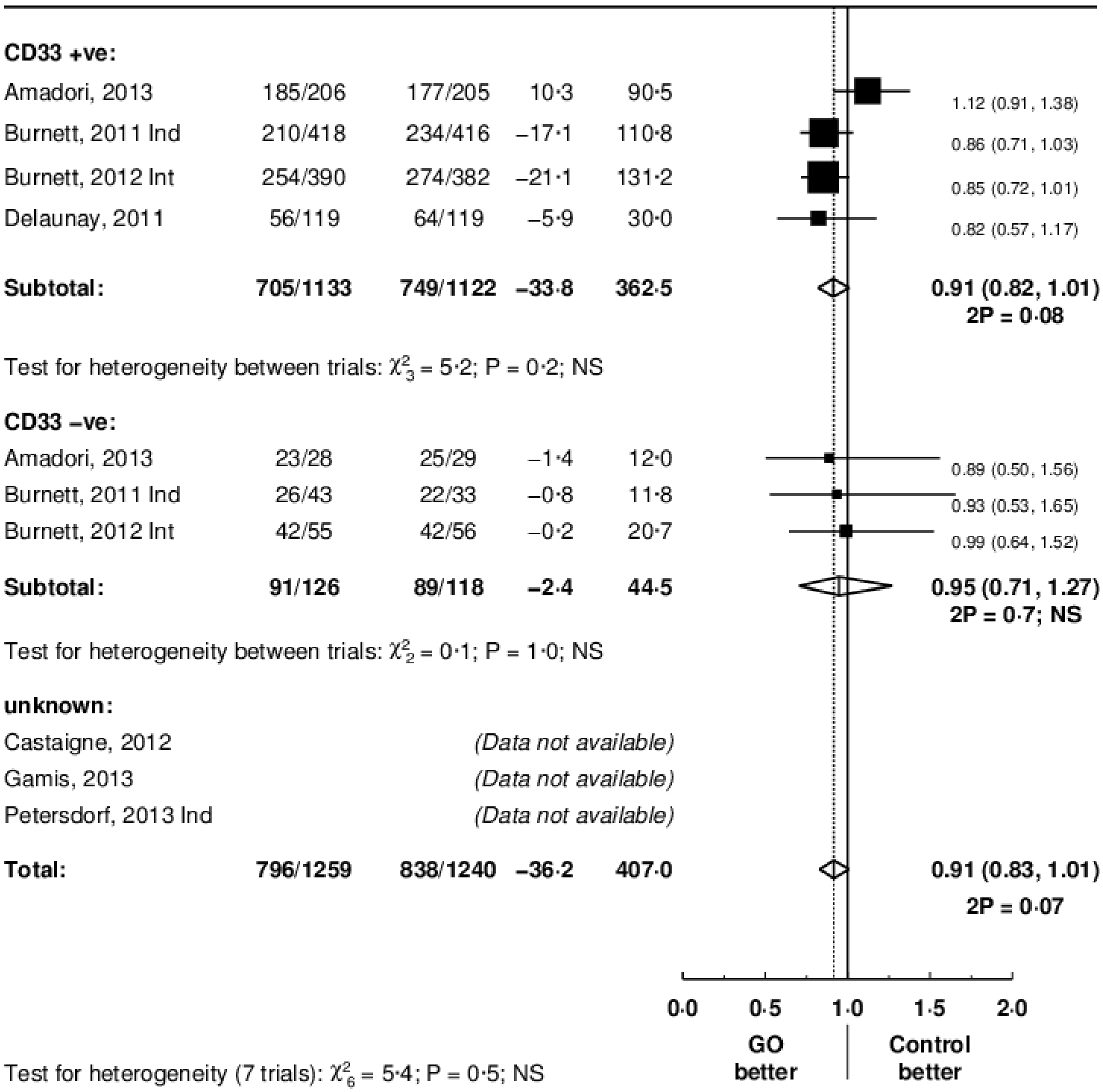

Test for heterogeneity (7 trials): $\chi^2_6 = 5·4$; P = 0·5; NS

Test for heterogeneity between subtotals: $\chi^2_1 = 0·1$; P = 0·8; NS

Reference List

(1) A Phase III Study of the Addition of Gemtuzumab Ozogamicin (Mylotarg®) Induction Therapy Versus Standard Induction with Daunomycin and Cytosine Arabinoside Followed by Consolidation and Subsequent Randomization to Post-Consolidation Therapy with Gemtuzumab Ozogamicin (Mylotarg®) or No Additional Therapy for Patients under Age 61 with Previously Untreated de novo Acute Myeloid Leukemia (AML). http://www.mhlw.go.jp/stf/shingi/2r9852000000vrz2-att/2r9852000000vs34.pdf. Accessed 25th March 2013..

Further Supplementary Data

Supplementary 1: Search filters

Supplementary 2: Available outcomes for each trial

Supplementary 3: Definitions of outcomes of trials in meta-analysis

Supplementary 4: Summary of Included Trial Characteristics and Quality

Supplementary 5: Details of trials from online trial databases

Supplementary 1

Search Filters

Cochrane Search Terms

Leukemia, Myelomonocytic, Acute OR exp Leukemia, Myeloid, Acute OR AML OR acute NEXT myel* NEXT leuk* OR acute NEXT granu* NEXT leuk* OR APML OR APL OR Acute NEXT Promyelo* NEXT Leuk* OR Acute NEXT Erythroblast* NEXT Leuk* OR Acute NEXT Myelomonocy* NEXT leuk* OR Acute NEXT Megakaryo* NEXT leuk* OR Acute NEXT Monocytic NEXT Leuk*

AND

gemtuzumab NEXT ozogamicin OR gemtuzumab OR Anti CD33 OR CD33 OR GO OR Mylotarg OR monoclonal NEXT antibod* OR calicheamicin

Embase Search Terms

1. AML.mp.

2. promyelocytic leukemia/ or promyelocytic leuk*.mp.

3. acute megakaryocytic leukemia/ or acute monocytic leukemia/ or acute myeloblastic leukemia/ or acute myelomonocytic leukemia/

4. acute granulocytic leukemia/

5. ((myelo$ or nonlympho$ or granulocytic$ or monocyt$ or megakaryo$ or promyelocyt$ or erythroblast$) and (leuk?em$ or leuc$)).mp.

6. erythroleukemia/

7. 1 or 2 or 3 or 4 or 5 or 6

8. monoclonal antibodies.mp. or monoclonal antibody/

9. CD33 antigen/ or CD33.mp.

10. gemtuzumab/ or gemtuzumab ozogamicin/ or gemtuzumab.mp.

11. mylotarg.mp.

12. GO.mp.

13. Anti CD33.mp.

14. calicheamicin derivative/ or calicheamicin.mp. or calicheamicin/ or calicheamicin gamma1/

15. 8 or 9 or 10 or 11 or 12 or 13 or 14

16. 7 and 15

17. limit 16 to (human and embase and (randomized controlled trial or controlled clinical trial or multicenter study or phase 2 clinical trial or phase 3 clinical trial or phase 4 clinical trial))

MEDLINE Search Terms

Cochrane based randomized control trial filter (Maximising sensitivity)

Terms for AML based on a Cochrane review [1]

1. exp LEUKEMIA, MYELOID, ACUTE/

2. acut$.tw.

3. ((myelo$ or nonlympho$ or granulocytic$ or monocyt$ or megakaryoblast$ or promyelocyt$ or erythroblast$) and (leuk?em$ or leuc$)).tw.

4. 2 and 3

5. erythroleuk?em$.tw.

6. (erythremic$ adj myelos$).tw.

7. LEUKEMIA, MYELOMONOCYTIC, ACUTE/

8. aml.tw.

9. or/4-8

10. 1 or 9

11. Antibodies, Monoclonal/ or Anti CD33.mp.

12. gemtuzumab ozogamicin.mp.

13. gemtuzumab.mp.

14. GO.mp.

15. Mylotarg.mp.

16. CD33.mp.

17. calicheamicin.mp.

18. 11 or 12 or 13 or 14 or 15 or 16 or 17

19. randomized controlled trial.pt.

20. controlled clinical trial.pt.

21. randomized.ab.

22. placebo.ab.

23. drug therapy.fs.

24. randomly.ab.

25. trial.ab.

26. groups.ab.

27. 19 or 20 or 21 or 22 or 23 or 24 or 25 or 26

28. exp animals/ not humans.sh.

29. 27 not 28

30. 10 and 18 and 29

Supplementary 2

Trial	Outcome								
	OS	RFS	DCR	CIR	ID	RD	CR	CRi	CRp
Amadori 2013	□	□	□	□	□	□	□		□
Burnett, 2011 Cons	□			□					
Burnett, 2011 Ind*	□	□	□	□	□	□	□	□	
Burnett, 2012 Int	□	□		□	□	□	□	□	
Burnett, 2012 NI	□	□					□	□	
Castaigne, 2012	□	□	□		□	□	□		□
Delaunay 2011**	□						□		
Fernandez, 2011	□	□	□	□					
Gamis 2013	□	□	□	□	□	□	□		
Hasle, 2012	□	□	□	□					
Lowenberg, 2010	□	□	□	□					
Petersdorf, 2013 Ind***	□	□				□	□	□	
Petersdorf, 2013 Maintenance		□	□	□					

*Up to date relapse and survival data from AML15 in AML16 publication used in analysis

**induction death defined as early deaths

*** CIR and DCR data from earlier interim report
CR = complete remission, CRi = Complete remission with incomplete haematological recovery, CRp = complete remission with platelet recovery.

Supplementary 3

Definitions of outcomes of trials in meta-analysis

CR	
**Trial name**	**Definition**
Amadori 2013	According to International Working Group (IWG) guidelines[2] (based on supplementary data).
Burnett, 2011	According to International Working Group (IWG) guidelines[2].
Burnett, 2012 Int	According to International Working Group (IWG) guidelines[2].
Burnett, 2012 NI	<5% leukaemic blasts on a normocellular bone marrow. Neutrophil recovery >1x10^9 /l and platelets to 100 x 10^9/l without evidence of extramedullary disease
Castaigne, 2012	<5% leukaemic blasts on a normocellular bone marrow. Neutrophil recovery >1x109 /l and platelets to 100 x 109/l
Delaunay 2011	Abstract only
Gamis 2013	<5% morphologic blasts (blasts) & extramedullary disease (EMD) resolved, from previous trial [3] Abstract only
Petersdorf, 2013 Ind	According to International Working Group (IWG) guidelines[2].

CRi	
**Trial name**	**Definition**
Amadori 2013	According to International Working Group (IWG) guidelines[2]. (CRp)
Burnett, 2011 Ind	According to International Working Group (IWG) guidelines[2]. (CRi)
Burnett, 2012 Int	According to International Working Group (IWG) guidelines[2]. (CRi)
Burnett, 2012 NI	As above but with insufficient recovery of counts (CRi)
Castaigne, 2012	According to International Working Group (IWG) guidelines[2]. (CRp)
Delaunay 2011	Abstract only
Petersdorf, 2013 Ind	According to International Working Group (IWG) guidelines[2]. (CRi)

Resistant disease	
**Trial name**	**Definition**
Amadori 2013	According to International Working Group (IWG) guidelines[2].
Burnett, 2011 Ind	Failure to eliminate disease including partial remission or death after 30 days without clinician evaluation.
Burnett, 2012 Int	According to International Working Group (IWG) guidelines[2].

Castaigne, 2012	According to International Working Group (IWG) guidelines[2]: no CR or CRp
Delaunay 2011	Abstract only
Gamis 2013	Based on induction death assumption (see below)
Petersdorf, 2013 Ind	According to International Working Group (IWG) guidelines[2].

**Induction death**	
Trial name	Definition
Amadori 2013	Not clearly defined.
Burnett, 2011 Ind	Death related to treatment and/or hypoplasia within 30 days or death within 30 days if no clinician's evaluation.
Burnett, 2012 Int	Not clearly defined.
Castaigne, 2012	Death during induction.
Delaunay 2011	Abstract only. Only described as early death.
Gamis 2013	Calculated from "2% in induction…with no difference by study arm".
Petersdorf, 2013 Ind	Induction toxicity

**Cumulative incidence of relapse (CIR)**	
**Trial name**	**Definition**
Amadori 2013	According to International Working Group (IWG) guidelines[2].
Burnett, 2011 Cons	According to International Working Group (IWG) guidelines[2].
Burnett, 2011 Ind	According to International Working Group (IWG) guidelines[2].
Burnett, 2012 Int	According to International Working Group (IWG) guidelines[2].
Castaigne, 2012	According to International Working Group (IWG) guidelines[2].
Delaunay 2011	Abstract only
Fernandez, 2011	Cumulative incidence analysis with death without relapse as competing event
Gamis 2013	"Relapse risk was defined as the time from end of course 1 for patients in CR to relapse or death because of progressive disease, where deaths from nonprogressive disease were considered competing events" from previous trial[3]. Termed RFS in text of abstract. Abstract only.
Hasle, 2012	Not clearly defined.
Lowenberg, 2010	"Competing risk of probabilities of relapse and death in first CR". "Relapse recurrence of leukemia after a first CR".
Petersdorf, 2013 Ind	For patients who achieve a CR and subsequently relapse (data from 2010 release[4])
Petersdorf, 2013 Maintenance	For patients in CR who relapse post randomisation to post consolidation treatment (from 2013 publication[5])

Cumulative incidence of Death in CR (CIDCR)	
**Trial name**	**Definition**
Amadori 2013	According to International Working Group (IWG) guidelines[2].
Burnett, 2011 Ind	According to International Working Group (IWG) guidelines[2].
Burnett, 2012 Int	According to International Working Group (IWG) guidelines[2].
Castaigne, 2012	Death in CR or CRp.
Delaunay 2011	Abstract only
Fernandez, 2011	Death without relapse
Gamis 2013	Death whilst in CR or "deaths from non progressive disease" from previous trial [3]. Abstract only
Hasle, 2012	Death in first CR
Lowenberg, 2010	Death in first CR
Petersdorf, 2013 Ind	For patients in CR who die without report of relapse (data from 2010 release[4])
Petersdorf, 2013 Maintenance	From 2013 publication[5]

RFS	
**Trial name**	**Definition**
Amadori 2013	According to International Working Group (IWG) guidelines[2]. From CR or CRp.
Burnett, 2011 Ind	According to International Working Group (IWG) guidelines[2]. From CR or CRi.
Burnett, 2012 Int	According to International Working Group (IWG) guidelines[2]. From CR or CRi.
Burnett, 2012 NI	Time from remission (CR or CRi) to either death or relapse, whichever first.
Castaigne, 2012	Time from CR or CRp to the date of relapse or death
Delaunay 2011	Abstract only
Fernandez, 2011	"Time from randomisation at the start of consolidation until relapse or death of any cause"
Gamis 2013	"Time from the end of course 3 (Intensification I) to death or relapse" (from http://clinicaltrials.gov/show/NCT00372593 accessed 03/12/2013). DFS from abstract text. Abstract only.
Hasle, 2012	Time from diagnosis until death in remission, relapse, second malignancy, or last follow-up,
Lowenberg, 2010	Time from post remission randomisation until relapse or death
Petersdorf, 2013 Ind	"Relapse free survival (RFS) was measured for patients who achieved CR from the day of CR until relapse or death from any cause, with the same censoring as DFS."
Petersdorf, 2013 Maintenance	"DFS was measured from the day of postconsolidation randomization until relapse

	from CR or death from any cause, whichever occurred first, with observation censored at the day of last contact for patients last known to be alive without report of relapse.”

**Cytogenetics subgroup analysis**					
**Trial name**	**Definition**	**Favourable**	**Intermediate**	**Adverse**	**Other**
Amadori 2013	EORTC criteria[6]	t(8;21), inv (16)	Normal or -Y	Chromosome 5 or 7 abnormalities or complex (>3 abnormalities)	All other abnormalities
Burnett, 2011	MRC criteria[7].	t(8;21), inv (16)	Normal, +8, +21, +22, del(7q), del(9q), Abnormal 11q23, all other	-5, -7, del (5q), Abnormal 3q, complex (5 unrelated cytogenetic abnormalities)	-
Burnett, 2012	MRC criteria[7].	As above	As above	As above	As above
Castaigne 2012	-	t(8;21), inv (16)	Normal, all other.	Monosomy 5, del(5q), monosomy 7, del(7q), t(6;11), t(9;22), 3q26 (except t(3;5), 11q23 (except t(9;11) and complex (>3).	
Delaunay 2011	GOELAMS – intermediate criteria only	t (15; 17), t (8; 21), inv (16)	Normal karyotype or Karyotype with other abnormalities , excluding the favourable group and the high risk group	-5/5q-, -7/7q-, t (9.22), t (6.9), 11q23 anomaly excluding the t (9; 11), abnormal 3q, complex karyotype (> 3 abnormalities)	
Petersdorf 2013	Presumably SWOG[8]	inv(16)/t(16;16)/del(16q) , t(15;17) with/without secondary aberrations; t(8;21) lacking del(9q) or	Normal, 18, 16, 2Y, del(12p)	del(5q)/25, 27/del(7q), abn 3q, 9q, 11q, 20q,	All other abnormalities

		complex karyotypes		21q, 17p, t(6;9), t(9;22) and complex karyotypes ($ 3 unrelated abn)	

**Diagnosis**	
**Trial name**	**Definition**
Amadori 2013	De Novo or Secondary (Therapy related or subsequent to preceding myelodysplasia)
Burnett, 2011	De novo or Secondary
Burnett, 2012 Int	De novo, Secondary and high risk MDS (>10% Blasts)
Castaigne, 2012	De novo only
Delaunay 2011	De novo only
Petersdorf, 2013 Ind	Previous haematological malignancy ineligible

**CD33 Positivity**	
**Trial name**	**Definition**
Amadori 2013	Above or below 20% CD33 expression[6].
Burnett, 2011	"CD33 expression status was determined in regional laboratories in accordance with national quality assurance methods". Defined as positive and negative Above or below 20% CD33 expression respectively.
Burnett, 2012 Int	Defined as positive and negative Above or below 20% CD33 expression respectively.
Delaunay 2011	“Expression of the CD33 antigen on the blasts was defined using standard method”[9].

**Median age of trial entrants**		
**Trial name**	**Median age of trial entrants**	**Above or below 60 years**
Amadori 2013	67	Above
Burnett, 2011 Cons	46	Below
Burnett, 2011 Ind	49	Below
Burnett, 2012 Int	67	Above
Burnett, 2012 NI	75	Above
Castaigne, 2012	62	Above
Delaunay 2011	50	Below
Fernandez, 2011	48	Below
Gamis 2013	9.9 (intervention arm)	Below
Hasle, 2012	Not reported	Below
Lowenberg, 2010	67	Above
Petersdorf, 2013 Ind	47	Below
Petersdorf, 2013 Maintenance	Not reported	Below

**Cumulative dose of GO**		
**Trial name**	**Cumulative dose of GO (mg/m2)**	**Above or equal vs below 9mg/m2**
Amadori 2013	12 (induction) 6 (consolidation)	Above
Burnett, 2011 Cons	3	Below
Burnett, 2011 Ind	3	Below
Burnett, 2012 Int	3	Below
Burnett, 2012 NI	4x 5mg flat dose	n/a
Castaigne, 2012	9 (induction) 6 (consolidation)	Above
Delaunay 2011	12	Above
Fernandez, 2011	6	Below
Gamis, 2013	6	Below
Hasle, 2012	10	Above
Lowenberg, 2010	18	Above
Petersdorf, 2013 Ind	6	Below
Petersdorf, 2013 Maintenance	15	Above

Supplementary 4: Summary of Included Trial Characteristics and Quality

Amadori 2013 [10]	
**Methods**	Randomised open label Phase III trial. Median follow up 5.2 years. Randomisation stratified to age, initial white blood cell count (WBC), Percentage with CD33 expression and institution.
**Population**	472 elderly patients 61-75 years. Performance status 0-2. Initial WBC less than 30x10^9/L. Median age 67. APML excluded. De novo or secondary AML. CD33 expression not a requirement for entry. 472 patients entered randomisation.
**Interventions**	GO randomisation: GO at 6 mg/m^2 on d ay 1 and 15 prior to a course of mitoxanthrone, cytarabine and etoposide (MICE) depending on disease progression or within 10 days of GO response assessment. At consolidation GO at 3 mg/m^2 at day -1 of each of two courses of ICE.<br>No GO randomisation: As above with GO.
**Outcomes**	Primary outcome- overall survival. Secondary outcome- CR/CRp/RFS/ Toxicity. Defined by International Working Group guidelines[2].

Risk of Bias

**Bias**	**Authors Judgement**	**Evidence for Judgement and comments**
Selection Bias	Low risk.	Central randomisation by minimisation stratification.
Performance Bias	Low risk.	Blinding not reported but outcome measurements are objective.
Attrition Bias	Low risk.	Outcome on 472 patients.
Reporting bias	Low risk.	Available outcomes in table 1. Intention to treat analysis.
Other bias	Low risk.	
**Overall Risk of Bias**	Low risk.	Unusual induction regimen administration timing, although this is discussed in the publication.

Burnett 2011: Induction randomisation[11]	
**Methods**	Randomised, controlled trial. Not blinded. Median follow-up 46 months.
**Population**	Adult less than 60 years of age. Initially older than 15, but later (2005) relaxed for paediatric patients as well. Median age 49. Primary or secondary AML. No APML. Previously untreated. 154 institutions in UK, Denmark and New Zealand. 2009 patients entered full trial, 1113 patients entered this stage of comparison. No CD33 criteria for entry.
**Interventions**	Induction stage. Assigned to Gemtuzumab Ozogomacin (GO) 3 mg/m^2 on day (D) 1 or not with one of three regimens: 1)Daunorubicin 50 mg/m^2 d1,3,5; cytarabine 100 mg/m^2 d1-10 every 12h 2)Daunorubicin 50 mg/m^2 d1,3,5; cytarabine 100 mg/m2 d1-10 every 12h; etoposide 100 mg/m2 d1-5 3)Fludarabine 30 mg/m^2 IV d2-6 inclusive, cytosine arabinoside 2 g/m^2 over 4h starting after fludarabine on d2-6, G-CSF (lenograstin 263 µg (1 vial) SC daily d1-7
**Outcomes**	Stated outcomes defined by [2], Stated outcomes not clearly defined initially but full reporting of all outcomes, see table 1. Updated data from AML16 report used in analysis [12].

Risk of Bias

Bias	Authors Judgement	Evidence for Judgement and comments
Selection Bias	Low risk.	Random sequence generation method not stated but allocated centrally by telephone (from protocol).
Performance Bias	Low risk.	Unblinded but outcomes objective, not subjective.
Attrition Bias	Low risk.	Follow up 97% complete.
Reporting bias	Low risk.	No clear omission of outcome data. Toxicity described but not fully profiled.
Other bias	Low risk.	
**Overall Risk of Bias**	Low risk.	

Burnett 2011: Consolidation Randomisation[11]		
**Methods**	As above	
**Population**	As above. Achieved complete remission after course 2. 2627 were in CR at this stage of trial but 948 patients entered this stage of randomisation for GO. Median age 46.	
**Interventions**	Consolidation stage (course 3) Assigned to Gemtuzumab Ozogomacin (GO) 3 mg/m^2 on day 1 together with one of three regimens; 1) MACE (amsacrine 100 mg/m^2 day 1-5; cytarabine 200 mg/m^2 continuous day 1-5; etoposide 100 mg/m^2 day 1-5) and MidAC (mitozantrone 10 mg/m^2 daily by slow IV push on day 1-5 inclusive (5 doses), cytosine arabinoside 1.0 mg/m^2 12-hourly by 2h IV infusion on day 1-3 inclusive (6 doses); 2) Ara-C 1.5 g/m^2 d1 given IV over 4h 12 hourly on day 1,3,5 (6 doses) 3) As before but Ara-C at 3g/m^2.	
**Outcomes**	As above.	
**Risk of Bias**		
**Bias**	**Authors Judgement**	**Evidence for Judgement and comments**
Selection Bias	Low risk.	As above. Significant drop out within consolidation phase of study prior to randomisation. Significant proportion of non-entrants had no reason given.
Performance Bias	Low risk.	As above.
Attrition Bias	Low risk.	Data not presented on 14 patients. Intention to treat analysis undertaken for those randomised to treatment.
Reporting bias	Low risk.	Results published on online supplemental data. No report on RFS. See table 1.
Other bias	Low risk.	
**Overall Risk of Bias**	Unclear risk.	Significant proportion of non-entrants to consolidation phase had no reason identified.

Burnett 2012 [12]	
**Methods**	Randomised Controlled Trial. Median follow up 30 months.
**Population**	1115 patients entered randomisation. De novo or secondary AML or high risk MDS

	with >10% blasts in bone marrow. CD33 expression not a requirement for entry. Patients over 60 years of age. Median age of trial participants 67 years of age.	
**Interventions**	GO on day 1 3mg/m^2 of first course and standard treatment, versus standard treatment alone. Standard treatment: Two courses of – (DA) Daunorubicin (50mg/m^2 D1,3,5 and cytarabine 100 mg/m^2 D1-10 every 12h; second course same dose of daunorubicin but cytarabine 100mg/m^2 d1-8 every 12 hours)or (D-Clo) Daunorubicin 50mg/m^2 d1,3,5 and clofarabine 20mg/m^2 d1-5. Could be randomised to a third course of DA (same dose of daunorubicin but cytarabine 100mg/m^2 d1-5 every 12 h) or not after CR in course 1 or CR in course 2 following PR in course 1. If ineligible for a reduced intensity transplant, randomisation to 9 courses of azacitidine 75mg/m^2 for 5 days every 6 weeks, or not as maintenance therapy.	
**Outcomes**	Primary outcome was overall survival. Secondary outcomes were CR, CR with incomplete recovery of counts, relapse free survival, death in remission and toxicity.	
**Risk of Bias**		
**Bias**	**Authors Judgement**	**Evidence for Judgement and comments**
Selection Bias	Low risk.	Randomisation method not reported. Central allocation by telephone (from protocol).
Performance Bias	Low risk.	Blinding not reported but outcome measurements are objective.
Attrition Bias	Low risk.	Follow up 96% complete.
Reporting bias	Low risk.	Full outcome reporting with toxicity profiling except for death in CR. See table 1.
Other bias	Low risk.	GO randomisation reflected 92% of entrants to the trial. Of the patients who did not enter randomisation reasons given. 2 patients withdrew consent before treatment and did not contribute to analysis, one on each arm. 96% compliance with GO treatment, 100% compliance with no GO treatment.
**Overall Risk of Bias**	Low risk.	

Burnett 2012 [13]		
**Methods**	Randomised open label Phase III trial. Median follow up 40 months.	
**Population**	Elderly patients unsuitable for intensive treatment. Median age 75. Included, primary and secondary AML and high risk MDS. CD33 expression not a requirement for entry. 495 patients entered randomisation.	
**Interventions**	Arm A: GO at 5mg flat rate, on day 1 of each course at up to 4 courses with low dose cytarabine as below.<br>Arm B: Standard therapy: Low dose cytarabine at 20mg twice a day by subcutaneous injection for 10days at approximately 6 week intervals.	
**Outcomes**	CR/CRi/RFS/ survival from remission and survival from relapse/OS	
**Risk of Bias**		
**Bias**	**Authors Judgement**	**Evidence for Judgement and comments**
Selection Bias	Low risk.	Randomisation method not reported. Central allocation by telephone (from protocol).
Performance Bias	Low risk.	Blinding not reported but outcome measurements are objective.

Attrition Bias	Low risk.	Outcome on 494 patients
Reporting bias	Low risk.	Full outcome reporting with toxicity profiling. See table 1.
Other bias	Low risk.	Dosing of GO results in variable GO doses when calculated by body weight. However, analysis suggests this did not affect outcomes.
**Overall Risk of Bias**	Low risk.	

Castaigne 2012[14]		
**Methods**	Randomised open label Phase III trial. Median follow up 14.8 months overall.	
**Population**	Adults aged 50-70. Previously untreated de novo AML. Median age of entrant 62.2. 280 patients entered randomisation. CD33 expression not a criteria for entry.	
**Interventions**	Arm A: GO 3mg/m^2 days 1,4,7 during induction and day 1 of each of the two consolidation courses with standard therapy.<br>Arm B: Standard therapy: daunorubicin (60mg/m2) i.v. days 1-3, cytarabine 200mg/m2 i.v. as continuous infusion for 7 days as induction. If not in CR, daunorubicin (60 mg/m2/day i.v. for 2 days and intravenous cytarabine (1000 mg/m2 per 12 h, infused over 2 h for 3 days). Consolidation: daunorubicin (60 mg/m2 i.v. for 1 day on first course or 2 days on second course), in combination with cytarabine (1000 mg/m2 i.v. per 12 h, days 1–4).	
**Outcomes**	Primary outcome was event free survival. Secondary outcomes CR or CR without full platelet recovery. Overall survival, relapse free survival and toxicity as well.	
**Risk of Bias**		
**Bias**	**Authors Judgement**	**Evidence for Judgement and comments**
Selection Bias	Low risk.	Computer generated randomisation with central allocation by telephone. Block stratification to centre and treatment. Block sizes of four.
Performance Bias	Low risk.	Open label study but objective outcomes required.
Attrition Bias	Low risk.	None lost to follow up. 2 withdrew consent and 1 death prior to induction. Intention to treat analysis.
Reporting bias	Low risk.	Full set of outcomes reported with toxicity profiling. See table 1.
Other bias	Low risk.	
**Overall Risk of Bias**	Low risk.	

Delaunay 2011 ASH (abstract only) [15]	
**Methods**	Randomised control trial between 2007-2010. Median follow up 20 months.
**Population**	De novo AML with an intermediate karyotype in patients aged 18-60. Median age 50. 254 patients involved. 238 patients analysed. CD33 antigen on blasts defined by standard method.
**Interventions**	Standard DA induction and MidAC intensive consolidation with randomisation with or without GO 6mg/m^2 at both treatment stages. Patients with European Leukemia Net (ELN) defined favourable molecular group received a second MidAc course followed by an autologous stem cell transplant. ELN intermediate 1 or 2 were

	considered for an allogeneic stem cell transplant. This was either a full myeloablative conditioned transplant preceded by a single course of chemotherapy or a reduced intensity regimen preceded by two courses of chemotherapy.
**Outcomes**	CR, OS, EFS and toxicity presented.

Risk of Bias

**Bias**	**Authors Judgement**	**Evidence for Judgement and comments**
Selection Bias	Unclear.	Unable to comment- abstract only.
Performance Bias	Low risk.	Open label- but unlikely to affect objective outcome measurements.
Attrition Bias	Unclear.	Unable to comment- abstract only.
Reporting bias	Unclear.	Unable to comment- abstract only. See table 1.
Other bias	Unclear.	Unable to comment- abstract only.
**Overall Risk of Bias**	Unclear.	Unable to comment- abstract only. Data extraction assumed equal representation in both groups as only 238 patients had been analysed to that stage. Therefore 238 patients split 1:1 between the two groups.

Fernandez 2011[16]	
**Methods**	Randomised Phase III, multicentre trial. Median follow-up 50.9 months.
**Population**	17-60 years of age. Primary AML without secondary AML or APML. Initially with CD33 positivity but later protocol amended for entry regardless of CD33 positivity. Median age of entrant was 48 for the intervention arm and 47 for the control arm. In first CR after induction therapy (cytarabine with standard or high dose daunorubicin and two cycles of high dose cytarabine). 270 patients randomised to treatment arms.
**Interventions**	Consolidation approach. Randomised to receive GO or not followed by autologous stem cell transplant. GO at a single dose of 6mg/m^2, followed by sargramostim 250 μ/m^2 until recovery of counts.
**Outcomes**	Disease free survival is the primary objective. Also interested in overall survival, cumulative incidence analysis with death without prior relapse as competing event.

Risk of Bias

**Bias**	**Authors Judgement**	**Evidence for Judgement and comments**
Selection Bias	Unclear risk.	Allocation and randomisation method not stated. Early closure of investigational arm and subsequent completion of trial by recruitment to standard consolidation treatment arm. However, these patients not included in analyses and the consolidation part of trial considered closed.
Performance Bias	Low risk.	Blinding not stated but objective outcomes stated.
Attrition Bias	Low risk.	All patients accounted for on an intention to treat analysis.
Reporting bias	Low risk.	Reported outcomes fully both on an intention to treat analysis as well as on secondary analysis of those who had received the autologous stem cell transplant. Toxicity discussion limited only to VOD. See table 1.

Other bias	Low risk.	Substantial number of patients did not proceed to an autologous stem cell transplant. However, they were accounted for and were evenly matched in both arms of treatment.
**Overall Risk of Bias**	Low risk.	

Gamis 2013 [17]		
**Methods**	Randomised Phase III, multicentre trial. Median follow-up 3.6 years for those alive.	
**Population**	0-29 years of age. Primary AML. CD33 positivity not required. Median age of entrant was 9.9 for the intervention arm and 9.5 for the control arm. 1022 patients of 1070 eligibile for analysis.	
**Interventions**	Randomised to standard therapy alone or addition of GO at a single dose of 3mg/m^2 at day 6 of induction I and on day 7 of intensification II as part of five cycles of chemotherapy. Risk stratification allowed patients at high risk to receive haematopoietic stem cell transplantation at end of intensification I.	
**Outcomes**	Event free survival and overall survival as the primary objective. Also interested in disease free survival, and induction remission rates.	
**Risk of Bias**		
**Bias**	**Authors Judgement**	**Evidence for Judgement and comments**
Selection Bias	Unclear risk.	Allocation and randomisation method not stated.
Performance Bias	Low risk.	Blinding open label but objective outcomes stated.
Attrition Bias	Unclear risk.	An intention to treat analysis. However, not all patients reported at this abstract stage.
Reporting bias	Unclear risk.	Reported outcomes fully both on an intention to treat analysis. Results which did not differ significantly between treatment arms not reported at abstract stage.
Other bias	Low risk.	
**Overall Risk of Bias**	Not applicable	Abstract only

Hasle 2012 [18]		
**Methods**	Randomised control trial. Median follow up was 4.2 years.	
**Population**	120 patients. Paediatric trial setting for patients with standard and high risk disease in CR1 post consolidation therapy, without HSCT due to lack of donor. Median age not given but age ranges included in text. CD33 expression not a requirement.	
**Interventions**	GO at 5mg/m^2 four weeks after last course of consolidation and repeated after three weeks.	
**Outcomes**	Toxicity, relapse rate and survival (OS and EFS.)	
**Risk of Bias**		
**Bias**	**Authors Judgement**	**Evidence for Judgement and comments**
Selection Bias	Unclear risk.	Randomisation method not specified in publication.
Performance Bias	Low risk.	Blinding not specified but outcomes measured were objective in nature.

Attrition Bias	Low risk.	Intention to treat analysis- one patients on no further therapy arm received GO, whilst three on the GO randomisation later withdrew consent and three relapsed.
Reporting bias	Low risk.	Full outcome reporting and toxicity profiling. See table 1.
Other bias	Low risk.	
**Overall Risk of Bias**	Low risk.	

Lowenberg 2010[19]		
**Methods**	Randomised, controlled trial, open-label. Median follow-up length 45 months. Multicentre study.	
**Population**	Older than 60 years of age. Primary AML and refractory anaemia with excess blasts. APML excluded. In first complete remission following two cycles of induction chemotherapy; one cycle of cytarabine and low or high dose daunorubicin, second cycle of cytarabine. No criteria for CD33 expression. Median age of patients 67 years. 232 evaluable and eligible patients randomised for post remission therapy.	
**Interventions**	Maintenance therapy. 3 cycles of GO at $6mg/m^2$ per 2 hour infusion at 4 week intervals versus no further treatment.	
**Outcomes**	Primary outcome- disease free survival (DFS). Secondary objectives, overall survival, DFS with failure either as a result of relapse or death in first CR, competing risk of relapse and death in first CR.	
**Risk of Bias**		
**Bias**	**Authors Judgement**	**Evidence for Judgement and comments**
Selection Bias	Unclear risk.	Randomisation were balanced by minimization according to site, AML vs MDS, induction therapy and stage of CR achievement. Sequence generation method not reported. Centrally allocated.
Performance Bias	Low risk.	Unblinded but outcomes objective, not subjective.
Attrition Bias	Low risk.	242 patients randomised but one patient lost to follow up and 9 had not attained CR. 232 reported. Significant number (52%) of patients reached CR but did not enter randomisation for accountable reasons. Mainly due to incomplete recovery from previous treatment, poor performance status or haematopoietic stem cell transplant.
Reporting bias	Low risk.	Reported OS, DFS, relapse probability and nonrelapse mortality. Toxicity profile reported only for GO arm of treatment, however other arm had not treatment. See table 1.
Other bias	Low risk	Only 65 patients out of the designated 113 patients completed all three cycles of treatment. Accepted issue with treating an elderly cohort of patients. Analysis was by intention to treat.
**Overall Risk of Bias**	Low risk	

Petersdorf 2013 [5]: Induction stage,		
**Methods**	Randomised, open label Phase III study. Multicentre. Median length of follow up not stated.	
**Population**	Patients with primary AML , APML excluded, age 18-60, previously untreated. Median age and CD33 expression criteria not reported. 637 randomised but 595 available for analysis (others ineligible or withdrew).	
**Interventions**	1 dose of GO at 6mg/m^2 on day 4 with daunorubicin (45mg/m2 Days 1, 2, 3); cytarabine (100mg/m2/d CI D1-7) versus No GO with a higher dose of daunorubicin (**60mg**/m2 Days 1, 2, 3); cytarabine (100mg/m2/d CI D1-7).	
**Outcomes**	Treatment outcomes defined by Cheson et al [2].but reported CR and CR with incomplete count recovery and median OS with relapse free survival.	
**Risk of Bias**		
**Bias**	**Authors Judgement**	**Evidence for Judgement and comments**
Selection Bias	Unclear risk.	Randomisation method not reported but patients stratified by age above and below 35.
Performance Bias	Low risk.	Open label study but outcomes are objective, not subjective.
Attrition Bias	Low risk.	Analysis of 595 patients, patients excluded from analysis were not eligible or withdrew from trial.
Reporting bias	Low risk.	DCR and CIR reported on interim supplement but not on full report.
Other bias	Low risk.	
**Overall Risk of Bias**	Unclear risk	The study was stopped early by a DSMC.

Petersdorf 2013 [5]: Post consolidation stage		
**Methods**	As above. 174 patients.	
**Population**	As above. Patients in trial who were in CR. Median age and CD33 expression criteria not reported.	
**Interventions**	Post consolidation GO, 3 doses at 5mg/m^2, every 28 days versus no additional therapy. Reported on 150 patients initially, data on 168 patients available subsequently.	
**Outcomes**	As above, DFS measured from the day of post consolidation randomisation until relapse from CR or death from any cause.	
**Risk of Bias**		
**Bias**	**Authors Judgement**	**Evidence for Judgement and comments**
Selection Bias	Unclear risk.	Randomisation stratified by cytogenetic risk and use of GO at induction. Method and allocation concealment not reported. Further more unclear as to why so few patients recruited to this phase study.
Performance Bias	Low risk.	Open label study but objective outcomes measured.
Attrition Bias	Low risk.	Data on 169 eligible patients. 5 registered patients who were not randomised were because they were ineligible.
Reporting bias	Low risk.	Relapsed free survival available. DCR present on interim release not available on full report See table 1.
Other bias	Low risk.	

**Overall Risk of Bias**	Unclear risk.	Trial stopped early by a DSMC. Unclear why so few recruited to this arm from the preceding consolidation phase.

Supplementary 5 Details of trials from online trial databases

Title	Sponsor	Phases	Enrollment	Other IDs	Start Date	Completion Date	Comment
Comparison of Two Treatments in Intermediate and High-risk Acute Promyelocytic Leukemia (APL) Patients to Assess Efficacy in 1st Hematological Complete Remission and Molecular Remission	Wyeth is now a wholly owned subsidiary of Pfizer	Phase 3	168	0903X-101128	May-02	Dec-07	Data not available for release.
Study of Chemotherapy in Combination With All-trans Retinoic Acid (ATRA) With or Without Gemtuzumab Ozogamicin in Patients With Acute Myeloid Leukemia (AML) and Mutant Nucleophosmin-1 (NPM1) Gene Mutation	University of Ulm	Phase 3	276	AMLSG 09-09	Feb-10	January 2020	Due for completion in 2014.
SCH 727965 in Patients With Acute Myelogenous Leukemia and Acute Lymphoblastic Leukemia (P04717AM2)(TERMINATED)	Schering-Plough	Phase 2	29	P04717	Jan-09	Apr-10	Terminated early
AML17: A programme of treatment development in younger patients with Acute Myeloid Leukaemia and high risk myelodysplastic syndrome	University of Cardiff	Phase III	-	AML17	Sept-08	Jul-14	Data not reported yet

Reference List

(1) Gurion R, Belnik-Plitman Y, Gafter-Gvili A et al. Colony-stimulating factors for prevention and treatment of infectious complications in patients with acute myelogenous leukemia. *Cochrane Database Syst Rev* 2012;6:CD008238.

(2) Cheson BD, Bennett JM, Kopecky KJ et al. Revised Recommendations of the International Working Group for Diagnosis, Standardization of Response Criteria, Treatment Outcomes, and Reporting Standards for Therapeutic Trials in Acute Myeloid Leukemia. J *Clin Oncol* 2003;21(24):4642-4649.

(3) Pollard JA, Alonzo TA, Loken M et al. Correlation of CD33 expression level with disease characteristics and response to gemtuzumab ozogamicin containing chemotherapy in childhood AML. *Blood* 2012;119(16):3705-3711.

(4) A Phase III Study of the Addition of Gemtuzumab Ozogamicin (Mylotarg®) Induction Therapy Versus Standard Induction with Daunomycin and Cytosine Arabinoside Followed by Consolidation and Subsequent Randomization to Post-Consolidation Therapy with Gemtuzumab Ozogamicin (Mylotarg®) or No Additional Therapy for Patients under Age 61 with Previously Untreated de novo Acute Myeloid Leukemia (AML). http://www.mhlw.go.jp/stf/shingi/2r9852000000vrz2-att/2r9852000000vs34.pdf. Accessed 25th March 2013

(5) Petersdorf SH, Kopecky KJ, Slovak M et al. A phase III study of gemtuzumab ozogamicin during induction and post-consolidation therapy in younger patients with acute myeloid leukemia. *Blood* 2013.

(6) Amadori S, Suciu S, Willemze R et al. Sequential administration of gemtuzumab ozogamicin and conventional chemotherapy as first line therapy in elderly patients with acute myeloid leukemia: a phase II study (AML-15) of the EORTC and GIMEMA leukemia groups. *Haematologica* 2004;89(8):950-956.

(7) Grimwade D, Walker H, Oliver F et al. The importance of diagnostic cytogenetics on outcome in AML: analysis of 1,612 patients entered into the MRC AML 10 trial. The Medical Research Council Adult and Children's Leukaemia Working Parties. *Blood* 1998;92(7):2322-2333.

(8) Slovak ML, Kopecky KJ, Cassileth PA et al. Karyotypic analysis predicts outcome of preremission and postremission therapy in adult acute myeloid leukemia: a Southwest Oncology Group/Eastern Cooperative Oncology Group study. *Blood* 2000;96(13):4075-4083.

(9) Efficacy of Gemtuzumab Ozogamycin for Patients Presenting an Acute Myeloid Leukemia (AML) With Intermediate Risk (LAM2006IR), http://clinicaltrials.gov/ct2/show/NCT00860639. Accessed 27th October 2013.

(10) Amadori S, Suciu S, Stasi R et al. Sequential Combination of Gemtuzumab Ozogamicin and Standard Chemotherapy in Older Patients With Newly Diagnosed Acute Myeloid Leukemia: Results of a Randomized Phase III Trial by the EORTC and GIMEMA Consortium (AML-17). *J Clin Oncol* 2013; 31(35):4424-30.

(11) Burnett AK, Hills RK, Milligan D et al. Identification of Patients With Acute Myeloblastic Leukemia Who Benefit From the Addition of Gemtuzumab Ozogamicin: Results of the MRC AML15 Trial. *J Clin Oncol* 2011;29(4):369-377.

(12) Burnett AK, Russell NH, Hills RK et al. Addition of Gemtuzumab Ozogamicin to Induction Chemotherapy Improves Survival in Older Patients With Acute Myeloid Leukemia. *J Clin Oncol* 2012; 30(32):3924-31.

(13) Burnett AK, Hills RK, Hunter AE et al. The addition of gemtuzumab ozogamicin to low-dose Ara-C improves remission rate but does not significantly prolong survival in older patients with acute myeloid leukaemia: results from the LRF AML14 and NCRI AML16 pick-a-winner comparison. *Leukemia* 2012; 27(1):75-81.

(14) Castaign e S, Pautas C, Terré C et al. Effect of gemtuzumab ozogamicin on survival of adult patients with de-novo acute myeloid leukaemia (ALFA-0701): a randomised, open-label, phase 3 study. *The Lancet* 2012;379(9825):1508-1516.

(15) Delaunay J, Recher C, Pigneux A et al. Addition of Gemtuzumab Ozogamycin to Chemotherapy Improves Event-Free Survival but Not Overall Survival of AML Patients with Intermediate Cytogenetics Not Eligible for Allogeneic Transplantation. Results of the GOELAMS AML 2006 IR Study. Presented at ASH 2011. *ASH Annual Meeting Abstracts* 2011;118(21):79.

(16) Fernandez HF, Sun Z, Litzow MR et al. Autologous Hematopoietic Cell Transplantation as Consolidation for Younger Patients with Acute Myeloid Leukemia in First Complete Remission: Follow-up of E1900. Presented at ASH 2009. *ASH Annual Meeting Abstracts* 2009;114(22):3405.

(17) Gamis A, Aplenc R, Alonzo TA et al. Gemtuzumab Ozogamicin (GO) In Children With De Novo Acute Myeloid Leukemia (AML) Improves Event-Free Survival (EFS) By Reducing Relapse Risk Results From The Randomized Phase III Childrens Oncology Group (COG) Trial, AAML0531. *Blood* 2013;122(21):355.

(18) Hasle H, Abrahamsson J, Forestier E et al. Gemtuzumab ozogamicin as postconsolidation therapy does not prevent relapse in children with AML: results from NOPHO-AML 2004. *Blood* 2012;120(5):978-984.

(19) Lowenberg B, Beck J, Graux C et al. Gemtuzumab ozogamicin as postremission treatment in AML at 60 years of age or more: results of a multicenter phase 3 study. *Blood* 2010;115(13):2586-2591.
